# IL-2 mutein selectively expands thymic memory Tregs with increased transendocytosis in healthy participants

**DOI:** 10.1172/jci.insight.193744

**Published:** 2026-09-17

**Authors:** Laura A. Cooney, Mitch Fahning, Liliane Khoryati, Anna Kus, Sheila Scheiding, Lori Blanchfield, Matt Lawrance, Basilin Benson, Kristina M. Harris, Gretchen A. Baltus, Shiuli Agarwal, Richard Wnek, Johannes F. Scheid, Kiki Cunningham-Bussel, Nancy D. Kim, S. Aubrey Stoch, Jyothsna Visweswaraiah, Nathan Higginson-Scott, Katalin Kis-Toth, Joanne L. Viney, Kevin L. Otipoby, Erik Sampson, Bridget Larkin, Daniel J. Campbell, S. Alice Long

**Affiliations:** 1 Immune Tolerance Network, University of Michigan, Ann Arbor, Michigan, USA.; 2 Benaroya Research Institute at Virginia Mason, Seattle, Washington, USA.; 3 Merck & Co., Inc., Rahway, New Jersey, USA.; 4 Pandion Therapeutics, Inc., a subsidiary of Merck & Co., Inc., Rahway, New Jersey, USA.; 5 Department of Immunology, University of Washington School of Medicine, Seattle, Washington, USA.

**Keywords:** Clinical Research, Immunology, Cytokines, Tolerance, Tregs

## Abstract

Tregs play an essential role in immune tolerance, and Treg-promoting therapies are in development for the treatment of many inflammatory disorders. IL-2–based therapies increase Treg frequency, but little is known about impacts on Treg heterogeneity and function. We extended analyses of an IL-2 mutein (MK-6194) single ascending dose trial in healthy human participants by comprehensively defining Treg subsets and gene expression changes in vitro and in vivo. We found highly specific and dose-dependent activation and expansion of Tregs in clinical and preclinical studies. Following a single subcutaneous dose in humans, thymus-derived Tregs were selectively activated and expanded, while peripherally induced Tregs were unaffected. Expanded Tregs had increased expression of genes and proteins consistent with activation, suppressor function, and homing to nonlymphoid tissue, as well as increased transendocytotic activity, as measured by CTLA-4–dependent capture of CD80 and CD86 from non-Tregs. These results shed light onto underlying mechanisms by which Treg-targeted therapy may promote immune tolerance.

## Abstract 2

The natural anti-inflammatory powers of Tregs are an attractive therapeutic target. We show treatment with engineered IL-2 mutein augments endogenous Treg function in new ways.

## Introduction

Tregs are an attractive therapeutic target, and strategies designed to manipulate Treg number, function, and specificity are under investigation for the treatment of autoimmune and inflammatory diseases (1, 2). Tregs are highly heterogeneous and can be categorized based on their origin as either thymus-derived “tTregs” that express Helios or peripherally induced “pTregs” that are Helios negative; activation state; suppressive function; and tissue-homing potential (3). Yet, how Treg-targeted therapies affect different types of Tregs in vivo is not fully understood (4).

Tregs depend on the cytokine IL-2 (5). The IL-2 receptor (IL-2R) is expressed as either an intermediate-affinity dimer consisting of 2 subunits, the β-chain (IL-2Rβ; CD122) and the common γ-chain (IL-2Rγc; CD132), or a high-affinity trimer that also includes the α-chain (IL-2Rα; CD25) (6, 7). The intermediate-affinity dimer is expressed at high levels by NK cells and memory T cells (8, 9), whereas the high-affinity trimer is constitutively expressed by Tregs, making these cells highly sensitive to low levels of IL-2 (10). Low doses of recombinant human IL-2 have been used in clinical trials to preferentially expand CD25^hi^ Tregs in patients with autoimmune disease with some therapeutic benefit (11). However, finding a dose that activates Tregs without activating other immune cells remains a challenge, and the short half-life of recombinant IL-2 in vivo necessitates frequent dosing (12).

MK-6194 is a modified IL-2 protein (IL-2 mutein) fused to the IgG Fc domain (13–15) with increased binding affinity for IL-2Rα (CD25) and reduced binding affinity for IL‑2Rβ (CD122) relative to wild-type IL-2. Thus, MK-6194 preferentially activates CD25^hi^ Tregs over NK cells and memory T cells that express the intermediate-affinity IL-2R dimer (12, 16). Results of single and multiple ascending dose trials of MK-6194 in healthy volunteers demonstrated that treatment was well tolerated and resulted in expansion of total Tregs without increased frequency of conventional T (Tconv) and NK cells (13). DNA methylation status of CpG sites in the FOXP3 Treg-specific demethylation region (TSDR) confirmed that these expanded Tregs were stably committed (13). To better understand therapeutic effects, we expanded on this previous report from Scheid et al. by assessing the relative responsiveness of Treg subsets to IL-2 mutein treatment in vivo, as well as characterizing effects on gene expression, protein expression, and in vitro transendocytotic activity of Tregs expanded in vivo in participants from the single ascending dose trial.

Here, we evaluate the selectivity and functionality of MK-6194 in vitro, in preclinical animal models, and in humans in a single ascending dose phase Ia clinical trial (13, 14). We show that MK-6194 stimulates CD25^hi^ Tregs, as measured by STAT5 phosphorylation, and preferentially expands Tregs in a humanized mouse model. In humans, MK-6194 preferentially expands activated thymus-derived memory tTregs without expanding peripherally induced pTregs. Expanded Tregs display increased expression of markers associated with activation, tissue homing, and suppressor function at both the transcript and protein levels. Moreover, Tregs from IL-2 mutein–treated participants demonstrate enhanced in vitro transendocytotic activity, mediated by CTLA-4–dependent capture of CD80 and CD86 from non-Treg cells.

## Results

Treg activation and expansion induced by IL-2 mutein is highly selective in preclinical models. To better define Treg expansion and MK-6194 specificity broadly defined in Scheid et al. (13), we first characterized MK-6194 selectivity in vitro by stimulating PBMCs from healthy donors, followed by flow cytometry for induction of STAT5 phosphorylation (pSTAT5). After stimulation with MK-6194 or recombinant wild-type IL-2 (0.25 pM to 20 nM) for 30 minutes, MK-6194 dose-dependently induced pSTAT5 in CD25^hi^ Tregs but was approximately 10-fold less potent than wild-type recombinant IL-2 (Figure 1A). In contrast, MK-6194 induced little to no response in CD25^lo^CD4^+^ Tconv cells or NK cells, whereas recombinant wild-type IL-2 induced pSTAT5 in all 3 cell types at higher doses (Figure 1A).

To explore the in vivo kinetics and selectivity of MK-6194, we used a NOD SCID gamma (NSG) mouse model reconstituted with human CD34^+^ hematopoietic stem cells (17). Reconstituted NSG mice received a single, subcutaneous dose of 3 μg MK-6194 (equivalent to approximately 0.15 mg/kg) or vehicle, and blood was collected on days 1, 3, 5, 6, 7, and 9 after treatment for immunophenotyping (Figure 1B). Consistent with human PBMCs, MK-6194 administration resulted in increased frequency of Tregs, peaking on day 7. In contrast, no significant changes in the frequencies of Tconv or NK cells were observed. A dose-response curve was also assessed in the humanized mouse model, comparing Treg expansion following a single dose of 0.04, 0.12, and 0.4 mg/kg of MK-6194 (Supplemental Figure 1; supplemental material available online with this article; https://doi.org/10.1172/jci.insight.193744DS1). Average Treg expansion increased linearly with increasing dose but was more heterogeneous at the highest dose. Furthermore, cynomolgus monkeys were administered a single subcutaneous dose of 0.5 mg/kg of MK-6194, and whole blood was collected on day 7 after treatment for immunophenotyping. Similarly to humanized mice, Tregs showed a significant expansion from baseline, whereas other potentially IL-2–responsive cell populations, including CD8^+^ T cells, NK cells, and central and effector memory CD4^+^ T cells, had little to no measurable change compared to baseline (Figure 1C).

To determine whether chronic autoimmune disease impacts the sensitivity and selectivity of the Treg response to MK-6194, PBMCs from healthy controls or donors with type 1 diabetes (T1D), systemic lupus erythematosus (SLE), or ulcerative colitis (UC) were stimulated with MK-6194 or wild-type IL-2 in vitro, and the response was measured by flow cytometry for pSTAT5. As shown in Figure 1D, MK-6194 induced similar levels of pSTAT5 in human Tregs, regardless of autoimmune status (no significant differences between participant groups by 2-way ANOVA). Similarly, autoimmune disease did not impact the Treg selectivity of MK-6194, as non-Treg populations, such as CD4^+^ memory, CD8^+^ memory, NK cells, and γδ T cells showed no difference in pSTAT5 induction following stimulation (Supplemental Figure 2). Thus, the presence of chronic inflammation did not alter proximal responses to MK-6194.

### A single dose of IL-2 mutein induces selective, dose-dependent Treg expansion in healthy human participants.

Humanized mouse models and nonhuman primates may not fully recapitulate responses in humans. Therefore, to assess the function and selectivity of MK-6194 in humans, healthy participants received a single subcutaneous injection in a phase Ia single ascending dose study, with fixed doses ranging from 1 to 10 mg (equivalent to approximately 0.01–0.15 mg/kg, 1 mg: *n* = 7, 3.5 mg: *n* = 6, 5 mg: *n* = 5, 7.5 mg: *n* = 5, 10 mg: *n* = 6, placebo: *n* = 7). Blood samples were obtained from participants 10 days before treatment (day –10), prior to treatment on day 1, and on days 8, 15, and 29 after treatment (Figure 2A). Samples from day –10 or prior to treatment on day 1 were used interchangeably to represent baseline based on sample availability. To assess the effects of treatment on the abundance of a wide range of cell subsets, PBMCs were immunolabeled with a 43-marker cytometry by time-of-flight (CyTOF) panel designed to identify immune cell populations, with a focus on IL-2–responsive cell types (Supplemental Table 5). Similar to previous findings (13), both the frequency and absolute number of total Tregs showed a dose-dependent expansion in the MK-6194–administered cohorts (Figure 2B). Absolute number of Tregs was highest on day 8 and remained significantly elevated above baseline through day 15 for all but the lowest dose of 1 mg (for day 15: 3.5 mg cohort, *P* < 0.001; 5 mg cohort, *P* = 0.022; 10 mg cohort, *P* < 0.001; see Supplemental Table 1). By day 29, both absolute Treg numbers and relative frequencies returned to baseline in all cohorts (Figure 2B, representative gating in Supplemental Figure 3A).

To closely examine the effects of IL-2 mutein on non-Tregs, we assessed relative frequencies and absolute numbers of other predicted IL-2–responsive cell types at baseline versus day 8 after treatment with MK-6194. The cell types analyzed included naive and memory CD4^+^ and CD8^+^ Tconv cells, T helper type 1 (Th1) cells, cytotoxic CD8^+^ T (Tc1) cells, CD56^hi^ and CD56^dim^ NK cells, γδ T cells, and innate lymphoid cells (representative gating in Supplemental Figure 3B). We found a small increase in absolute numbers of CD56^hi^ NK cells only in the highest-dose cohort and a small decrease in Tc1 only in the lowest-dose cohort (Figure 2C, participant-level data in Supplemental Figure 4). Changes in relative frequencies of lymphocyte populations were also examined using unbiased clustering of CyTOF data (clustering details in Supplemental Figure 5A). As with hypothesis-based gating, we found a significant, dose-dependent increase in the relative frequency of the Treg cluster (metacluster 8, CD25^+^FOXP3^+^CD127^lo^) from baseline to day 8 (Figure 2D). There were no significant effects on the relative frequency of any other cell cluster at any dose (Figure 2D). Thus, of all lymphocyte subsets, only Tregs showed consistent, dose-dependent changes in absolute numbers and relative frequencies following treatment.

Although we ruled out significant effects of MK-6194 on the frequency of non-Treg populations, we also wanted to confirm that there were no significant effects on the phenotype of other IL-2–responsive cells, such as increased activation or inflammatory signaling that might undermine therapeutic effects of Treg expansion. To assess the transcriptional profile of IL-2–responsive cell populations other than Tregs, lymphocytes expressing high levels of CD122 (IL-2Rβ) were sorted from the 10 mg group at baseline and day 8 for single-cell RNA sequencing (scRNA-seq; representative gating in Supplemental Figure 6). Analysis of RNA expression profiles via uniform manifold approximation and projection (UMAP) and Louvain clustering identified 10 distinct cell clusters (Figure 3A). Expression of lineage-defining genes such as *CD3D*, *NCAM1*, *TCRGC2*, and *TRAC* identified the majority of these cells as NK or T cells (Figure 3B). Most T cells analyzed were either CD8^+^ αβ T cells or γδ T cells. Tregs are generally CD122^lo^ and thus were not captured in large enough numbers by our sorting strategy to permit analysis of Tregs in this dataset (Figure 3C, Supplemental Figure 6). Consistent with our CyTOF analyses, there were no significant changes in the relative abundance of any of the CD122^hi^ cell clusters (Figure 3D). Similarly, differential gene expression analyses using pseudobulk data found no significant changes in gene expression in any of the 10 clusters after MK-6194 treatment (Figure 3E). Thus, these data confirm the Treg selectivity of MK-6194, with limited effects on other IL-2–responsive non-Treg populations, including NK cells, CD8^+^ T cells, and γδ T cells.

### IL-2 mutein treatment results in prolonged IL-2R signaling in Tregs.

To examine proximal signaling of the IL-2R following MK-6194 treatment, we used flow cytometry to measure pSTAT5 in PBMCs isolated from study participants. PBMCs were immunolabeled immediately upon thawing with no additional stimulation in vitro, and thus pSTAT5 levels represent activation that occurred in vivo (Figure 4A). MK-6194 administration resulted in a dose-dependent increase in pSTAT5 levels in Tregs on day 8, which returned to baseline by day 15 (Figure 4B, representative gating in Supplemental Figure 7). Moreover, there was no detectable pSTAT5 increase in CD56^hi^ NK cells, CD4^+^ memory, or CD8^+^ memory T cells on day 8 (Figure 4C), confirming the Treg specificity, as described above.

Prolonged signaling through the IL-2R is consistent with the extended half-life of MK-6194 and may have important downstream effects on Treg phenotype and function (13, 14). However, prolonged stimulation through some cytokine receptors results in receptor desensitization (18), and therefore we asked whether prior treatment with IL-2 mutein may impact Treg responsiveness to restimulation with IL-2 in vitro. PBMCs isolated from MK-6194–treated participants were restimulated with recombinant wild-type IL-2 in vitro, followed by staining for pSTAT5 by flow cytometry (Figure 4D, representative staining in Supplemental Figure 7B). At all doses and time points examined, MK-6194 administration had no significant impact on the ability of expanded Tregs to reactivate pSTAT5 in vitro, indicating that the IL-2R remains functionally responsive after IL-2 mutein stimulation in vivo (Figure 4E).

### Thymus-derived memory Tregs are preferentially expanded in response to IL-2 mutein.

To determine whether MK-6194 preferentially expands specific Treg subsets, we performed unbiased clustering of CyTOF data within the Treg population (gating in Supplemental Figure 3A). UMAP visualization shows 2 clusters of Helios^+^ memory tTregs, both lacking CD45RA and CCR7 and expressing high levels of CD25 and CCR4 (Figure 5A). One of these clusters expressed high levels of the skin-homing receptor CLA (henceforth referred to as CLA^+^ tTreg), and the other expressed high levels of the co-inhibitory receptor TIGIT (henceforth referred to as TIGIT^+^ tTreg). We also identified 1 Helios^+^CD45RA^+^ naive tTreg subset and 1 Helios^–^ pTreg subset (Figure 5A, additional clustering detail in Supplemental Figure 8). Changes in the absolute numbers of these subsets on day 8 showed dose-dependent expansion of both CLA^+^ and TIGIT^+^ populations of memory tTregs, whereas there was no significant expansion of the naive tTreg or pTreg populations at any dose (Figure 5B).

Although both CLA^+^ and TIGIT^+^ subsets of memory tTregs expanded in response to IL-2 mutein, there were differences between the two. Firstly, CLA^+^ tTregs were approximately twice as frequent as TIGIT^+^ tTregs at baseline (average 10.4 ± 4.6 SD versus 5.56 ± 3.1 SD cells × 10^6^/L, respectively; *P* < 0.0001 by unpaired *t* test, *n* = 28, includes all cohorts). Secondly, TIGIT^+^ tTregs underwent approximately twice as much expansion as CLA^+^ tTregs (average 7.4 ± 6.8 SD versus 3.1 ± 1.3 SD fold increase, respectively; *P* = 0.0152 by unpaired *t* test, *n* = 17, includes 3.5 mg, 5 mg, and 10 mg cohorts). These observations raise the question of differential responsiveness to MK-6194 between Treg subsets. We predicted that response to treatment may be associated with higher surface levels of CD25 at baseline. However, the TIGIT^+^ tTreg subset expressed lower levels of CD25 and the cell proliferation marker Ki-67 at baseline compared with CLA^+^ tTregs (Figure 5C). Both memory tTreg subsets upregulated CD25 and Ki-67 expression on day 8 after treatment. Similarly to TIGIT^+^ tTregs, naive tTregs expressed low levels of CD25 and Ki67 at baseline and responded to IL-2 mutein treatment with a similar upregulation of CD25 and Ki67; however, unlike TIGIT^+^ tTregs, naive tTregs did not increase in absolute number (Figure 5B). Thus, MK-6194 activates both naive and memory tTregs but preferentially expands memory tTregs, with the TIGIT^+^ subset having greater proliferative potential associated with lower expression of activation markers at baseline. In contrast to memory tTregs, pTregs did not respond to MK-6194, showing no increase in abundance and no increase in CD25 or Ki67 expression, consistent with low CD25 expression at baseline (Figure 5C).

To determine whether the differential responsiveness between tTregs and pTregs begins at the level of IL-2R signaling, we examined the induction of pSTAT5 by naive tTregs (CD45RA^+^Helios^+^), memory tTregs (CD45RA^–^Helios^+^), and pTregs (CD45RA^–^Helios^–^) following in vitro stimulation with wild-type IL-2 or MK-6194. We found that although all Treg subsets showed STAT5 phosphorylation in response to MK-6194, responses in pTregs and naive tTregs were weaker than memory tTregs (Figure 5D). Thus, the selective expansion of memory tTregs after MK-6194 treatment may be due in part to enhanced responsiveness at the level of the IL-2R signaling.

### IL-2 mutein administration results in transient transcriptional changes in circulating Tregs.

To examine the transcriptional responses of Tregs to MK-6194 treatment, we performed bulk RNA-seq on CD4^+^CD25^+^CD127^lo^ Tregs sorted from participants from the 1 mg (*n* = 5), 3.5 mg (*n* = 6), and 10 mg (*n* = 4) dosing cohorts at baseline and on days 8, 15, and 29 after treatment (representative sort gating in Supplemental Figure 9). Analysis identified 246 differentially expressed genes (DEGs) whose expression changed (log_2_[fold change] > 1, FDR < 0.05) following MK-6194 treatment (Figure 6A). The largest number of transcriptional changes was observed in the 10 mg dose cohort on day 8 (166 DEGs), with approximately half as many changes in the 3.5 mg dose cohort (82 DEGs) and few changes in the 1 mg cohort (13 DEGs) at the same time point. Consistent with the robust Treg expansion, DEGs included those regulating cell cycle progression such as *CSK2*, *CDC25C*, *CDC45*, *CDKN2A*, *CDKN2B*, and *RBBP8*, as well as genes controlling cell death, such as *CASP4*, *FAS*, and *DAD1*. DEGs also included genes that regulate cell signaling (*PRKCB*, *AKT3*, *JUN*, *JUNB*, *JUND*, *FOSB*, *DGKE*, *MAPKAP3*, and *CD3D*), migration (*CCR7*, *SELL*, *CCR5*, *ITGAM*, and *CCR3*), and cytokine responses (*TGFBR2*, *IL1RL1*, *LTB*, *IL2RG*, and *IL32*), as well as transcription factors that control Treg differentiation and function (*TCF7*, *BHLHE40*, *MAF*, *MYB*, and *TBX21*).

Based on changes in gene expression observed in the 10 mg dose cohort, 4 subsets of DEGs were identified (Figure 6, A and B): (a) downregulated genes (45 genes); (b) genes transiently upregulated on day 8 that returned to baseline on day 15 (36 genes); (c) persistently upregulated genes with elevated expression on days 8 and 15 (128 genes); and (d) late genes whose expression increased on day 15 (36 genes). Nearly all changes in gene expression returned to baseline in all cohorts by day 29. As expected, IL-2 mutein treatment increased expression of canonical Treg and IL-2R/STAT5 target genes, including *FOXP3*, *IL2RA* (CD25), *SOCS2*, *BCL2*, and *MYB*, among others (Figure 6C), and gene set enrichment analysis showed significant enrichment of genes in the IL2 STAT5 signaling hallmark gene set on day 8 compared with baseline in the 10 mg dose cohort (Figure 6D). FOXP3 and CD25 are both transiently expressed following activation of human CD4^+^ Tconv cells, which can introduce challenges in distinguishing Tregs from activated Tconv cells. Therefore, to confirm that the CD4^+^CD25^+^CD127^lo^ cells sorted following MK-6194 treatment consisted of bona fide stably committed Tregs, we examined expression of previously published “activation-independent” gene sets that reliably distinguish human Treg and Tconv populations (19). Indeed, we found that MK-6194 treatment significantly increased expression of the Treg gene signature (*P* < 0.001), whereas expression of the Tconv gene signature was low at baseline and did not significantly change after administration (Figure 6E), confirming at the transcriptional level that expanded CD25^+^ T cells are true Tregs and not activated CD25^+^ Tconv cells.

Among MK-6194–induced DEGs, Gene Ontology (GO) term enrichment identified pathways involved in cellular activation and proliferation, with upregulated pathways including “positive regulation of cell activation,” “regulation of cyclin-dependent protein kinase activity,” and “negative regulation of intrinsic apoptotic signaling pathway” (Figure 6F). Upregulated DEGs were also enriched in genes associated with the “oxidative phosphorylation,” “ATP metabolic processes,” and “proton membrane transport” pathways, indicating that MK-6194–expanded Tregs have undergone metabolic reprogramming to support their robust expansion and function. In contrast, DEGs downregulated by MK-6194 treatment were enriched in genes from the “negative regulation of mitotic cell cycle,” “T cell differentiation in the thymus,” and “regulation of RNA metabolic processes” pathways.

### Treg expression of markers of suppressive function and tissue homing are enhanced by IL-2 mutein administration.

In addition to controlling Treg abundance, IL-2R signaling regulates Treg activation state and immunosuppressive function (20). Indeed, we found elevated expression of genes associated with Treg activation and suppressor function such as *CTLA4*, *ICOS*, *LRRC32* (GARP), *TIGIT*, *HAVCR2* (TIM-3), *IL1R2*, *TNFRSF4* (OX-40), *MKI67*, *TNFRSF9* (41BB), and *TNFRSF18* (GITR) on day 8 after 10 mg MK-6194 treatment (Figure 7A).

Tregs acquire different phenotypes and functions depending on where they reside (3), such as lymphoid tissue (LT) versus nonlymphoid tissue (NLT), and the disease state, such as healthy tissues versus solid tumors or sites of autoimmune inflammation. To assess the effects of MK-6194 on Treg adaptation to various human tissues and disease states, we performed gene set enrichment analysis of transcriptional changes induced by IL-2 mutein compared with 4 consensus human Treg gene sets (21): “High in diseased NLT” (based on 10 tumor gene sets and 2 synovial fluid gene sets), “Low in diseased NLT” (based on 7 gene sets comparing blood, healthy tissue, and diseased NLT), “Combined healthy NLT” (based on 9 gene sets including uterus, colon, skin, fat, and other tissues), and “Combined healthy LT” (based on 5 gene sets from spleen, lymph node, blood, and colon-associated LT). We found that the transcriptional signatures of Tregs from both healthy and diseased NLT were significantly enriched in MK-6194–expanded Tregs, whereas genes that were low in Tregs from diseased NLT were significantly enriched in the baseline Tregs prior to MK-6194 treatment (Figure 7B). Conversely, MK-6194 treatment had no effect on enrichment of the healthy LT Treg signature. Thus, MK-6194–expanded Tregs upregulate a gene signature consistent with residency and function in NLTs.

To confirm that these transcriptional changes in Tregs were also reflected at the protein level, we analyzed expression of markers of activation, suppressor function, and migration by CyTOF (Figure 7C). This analysis confirmed transiently increased protein expression of the activation and proliferation markers Ki-67, ICOS, and OX-40, as well as increased protein expression of CTLA-4, CD39, and TIGIT, suggesting elevated capacity for Treg suppressor function (22–25). Furthermore, the homing receptors α4β7 integrin and CXCR3 that direct Tregs to inflamed NLTs were upregulated following treatment, whereas the LT homing receptor CCR7 was downregulated, further suggesting preferential homing to NLT over LT.

In a previous study analyzing murine Tregs after treatment with a Treg-selective murine IL-2 mutein (26), we found that elevated expression of CTLA-4 was associated with enhanced ability to strip the co-stimulatory ligands CD80 and CD86 from the surface of antigen-presenting cells (APCs) via a process termed transendocytosis. This is an important Treg function that supports immune tolerance by reducing the ability of APCs to activate Tconv cells (25). Given the similar upregulation of CTLA-4 expression in MK-6194–expanded Tregs (Figure 7, A and C), we used an in vitro system to measure CTLA-4–dependent transendocytosis of CD80 and CD86 (Figure 7D). CD4^+^ T cells were isolated from 4 participants at baseline and day 8 after treatment with 7.5 mg MK-6194. Total CD4^+^ T cells were co-cultured for 6 hours with NIH3T3 cells transfected with human CD86-GFP and CD80-mCherry fusion proteins in the absence of further stimulation. Non-adherent cells were analyzed by flow cytometry to identify FOXP3^+^ Tregs that obtained GFP and mCherry fluorescence via transendocytosis (Figure 7D). We found that MK-6194 treatment significantly increased ligand capture by Tregs, as evidenced by increased frequency of GFP^+^mCherry^+^ cells, which was inhibited by addition of a CTLA-4–blocking antibody to the co-culture (Figure 7, E and F). By contrast, FOXP3^–^ Tconv cells in the same cultures showed minimal ability to capture CD80 and CD86. Taken together, these results suggest that MK-6194–expanded Tregs are poised to migrate to NLTs and strip APCs of key costimulatory molecules.

## Discussion

IL-2–based therapy to promote Treg expansion and function is a strategy to treat autoimmune and inflammatory disorders (1, 2). However, these efforts have been hampered by the short half-life of wild-type IL-2 and evidence of activation of Tconv, γδ T cells, and NK cells (20, 27, 28). Additionally, how heterogeneous populations of human Tregs differentially respond to signals through IL-2R in vivo warrants further study.

Low-dose IL-2 therapy expanded Tregs in patients with T1D, but it also expanded cytotoxic T cells and NK cells (27, 29). Similarly, in individuals with SLE, low-dose IL-2 induced proliferation of NK cells and CD4^+^ and CD8^+^ Tconv cells (20). Use of rapamycin in combination with low-dose IL-2 to inhibit mTORC1 signaling can enhance Treg selectivity but may also compromise Treg function, as mTORC1 activation is required for full suppressive activity (29, 30). In fact, rapamycin/IL-2 combination therapy in patients with T1D increased Treg frequency but resulted in a transient β cell dysfunction (29), limiting the potential utility of this approach. By contrast, we found no significant transcriptional changes or evidence of IL-2R signaling in non-Treg cells after MK-6194 administration in vivo, even at the highest dose tested, clearly demonstrating increased specificity over low-dose IL-2 therapy.

The strength and duration of IL-2R signaling have downstream effects on Treg phenotype and function, which may be an important factor in the clinical efficacy of modified IL-2 therapies (31). Interestingly, pSTAT5 activation was detectable ex vivo for at least 1 week after a single administration of MK-6194, even at the lowest dose (1 mg), at which point drug levels in the circulation were nearly undetectable (13). This persistent stimulation may have important implications for treatment of autoimmune disease such as SLE and T1D, where Tregs have decreased IL-2R sensitivity (32–34). The prolonged activation of IL-2R by MK-6194 allows for robust activation of Tregs, as evidenced by the upregulation of TCR-dependent activation genes such as *TNFRSF9*, *ICOS*, and *CTLA4*, similar to the upregulation of a TCR-dependent gene signature observed in mice following IL-2 mutein treatment (26). IL-2R signaling may directly synergize with TCR signaling by converging on key signaling hubs such as mTORC1 that control Treg activation and proliferation (35), or by promoting adhesion to APCs to promote TCR signaling (10). Importantly, Tregs from MK-6194–treated participants induced robust STAT5 phosphorylation upon restimulation with wild-type IL-2 in vitro, suggesting that negative regulatory circuits that may limit subsequent IL-2 responsiveness were not activated.

We found a hierarchy of responsiveness among Treg subsets: pTregs were the least responsive. They showed reduced induction of pSTAT5 after in vitro stimulation with MK-6194, and appeared unresponsive in vivo, as suggested by lack of upregulation of CD25 or Ki67 and lack of proliferation. Among tTregs, both naive and memory subsets induced pSTAT5 after in vitro stimulation and responded to MK-6194 in vivo by upregulating CD25 and Ki67, but the absolute number of naive tTregs remained constant, while memory tTregs expanded. The lack of expansion of naive tTregs despite increased expression of the proliferative marker Ki-67 likely reflects their conversion into memory phenotype tTregs (36, 37). In addition, the selectivity of MK-6194 for cells with high CD25 expression is demonstrated by the lack of expansion of Helios^–^ pTregs, which express CD25 at lower levels than naive or memory tTregs. Selective in vivo expansion of Helios^+^ tTregs but not pTregs also suggests that the expanded Treg population consists of bona fide, stably committed Tregs that are unlikely to dedifferentiate into Tconv cells. This conclusion is supported by the increased expression of a Treg-specific gene expression signature and decreased expression of an activated Tconv cell signature (Figure 6E), as well as by the increase in demethylation of the FOXP3 Treg-specific demethylation region (TSDR) in expanded Tregs following in vivo administration of MK-6194 (13). Our findings are consistent with results from clinical trials of low-dose IL-2 and a modified IL-2 containing polyethylene glycol (PEGylated IL-2), which demonstrated selective expansion of Helios^+^ tTregs (38, 39).

Clustering of our CyTOF data uncovered memory tTregs that were further separated into 2 predominant subsets: one expressing high levels of activation markers as well as the skin-homing receptor CLA, and the other with less activation but increased levels of the co-inhibitory receptor TIGIT, both of which expanded in response to MK-6194 administration. Interestingly, the less activated TIGIT^+^ subset underwent greater expansion, suggesting that pre-existing Treg activation may limit rather than enhance responsiveness to IL-2 mutein. Relative expansion of TIGIT^+^ versus CLA^+^ memory tTreg subsets may have therapeutic implications. CLA^+^ Tregs are linked to inflammatory skin disease (40–42) and TIGIT^+^ Tregs are known to be highly activated, stably committed, and highly suppressive (22, 23). Another group has shown that low-dose IL-2 therapy induced proliferation of CLA^+^ Tregs in the blood, resulting in increased Treg abundance in the skin of patients with SLE (20). Thus, expansion of CLA^+^ Tregs may represent an important component of clinical efficacy when IL-2 muteins are applied to cutaneous diseases. In fact, treatment with PEGylated IL-2 led to a dose-dependent reduction in the severity of atopic dermatitis, psoriasis, and cutaneous lupus (38, 43). Further clinical studies are warranted to determine the relationship between selective expansion of CLA^+^ Tregs by IL-2 muteins and clinical efficacy in cutaneous diseases. Expansion of TIGIT^+^ Tregs by other IL-2–based therapies has not been previously described, and further comparative studies are warranted.

Consistent with increased activation, we found that known markers of Treg function such as CTLA-4, GARP, and CD39 were upregulated after MK-6194 administration. We also found that the ability of Tregs to strip cells of the co-stimulatory ligands CD80 and CD86 via CTLA-4–dependent transendocytosis was enhanced by MK-6194 treatment, mirroring the effects of IL-2 mutein treatment in mice (26). Similarly, in a mouse model of skin graft rejection, treatment with IL-2 mutein led to increased Treg suppressor function, which was associated with increased Treg expression of CTLA-4, and a blocking antibody against CTLA-4 impaired the ability of IL-2 mutein to prevent graft rejection (44), but the role of transendocytosis was never examined in this model. Thus, suppression of co-stimulation via elevated CTLA-4 function may be an important and underappreciated immunosuppressive mechanism in the context of IL-2 mutein therapies.

In addition to regulating Treg activation and proliferation, genes associated with oxidative phosphorylation were significantly enriched in Tregs from MK-6194–administered participants, consistent with reports of low-dose IL-2 in patients with SLE (20). Metabolic pathways play an active role in controlling Treg differentiation, activation, and proliferation (45), and oxidative phosphorylation confers Tregs with flexibility in fuel choice and a survival advantage (46). Mitochondrial respiration is required for proper Treg function (47), and induction of the oxidative phosphorylation pathway is another way by which IL-2–based therapies may boost Treg function, but this link has not been directly tested here.

Our study comes with limitations. The limited availability of samples from this early-phase clinical trial precluded classical assessments of Treg function, such as suppression of bystander T cell proliferation. Although we observed similar in vitro responses to MK-6194 by PBMCs from healthy controls and patients with SLE, T1D, and UC, it is possible that this will not be representative of the in vivo response to MK-6194 in patients with inflammatory or autoimmune disorders. Polymorphisms in the *IL2RA* and *IL2RB* loci that may impact expression and signaling of the IL-2R are linked to multiple autoimmune diseases (11) and could alter responses to MK-6194. Additionally, upregulation of CD25 by activated CD4^+^ and CD8^+^ T cells may reduce Treg selectivity of MK-6194 in inflamed tissue sites. Further evaluations of in vivo Treg responses in autoimmune disease patients receiving IL-2 mutein therapy and the relationship with clinical outcomes will be important. Further studies of the relationship between the phenotypic changes described here and clinical outcomes are needed.

In summary, our results show that the IL-2 mutein MK-6194 is highly selective for Tregs. Among Treg subsets, only Helios^+^ tTregs expanded, with the greatest degree of response among tTreg subsets with an intermediate activation state. We further determined that expanded Tregs expressed markers that have been previously associated with activation, suppressor function, and tissue-homing potential. Together, these results expand our understanding of underlying mechanisms mediating effects of Treg-targeting drugs and support further clinical development.

## Methods

### Sex as a biological variable.

In animal studies, all mice were female, while nonhuman primates were 50% male and 50% female.

In the single ascending dose trial in healthy volunteers, participants were 62.5% male and 37.5% female (13). Sex was not considered as a biological variable.

### Clinical study design.

Details of the clinical study design and outcomes are described elsewhere (13). Briefly, this study was a first-in-human, randomized, double-blind, placebo-controlled, single ascending dose trial conducted in a single center aimed at understanding the effects of MK-6194 in Tregs in healthy participants (*N* = 56). Study participants were randomized 3:1 into MK-6194– or placebo-administered groups and split into 5 dose cohorts, including 1, 3.5, 5, 7.5, and 10 mg. After adjusting for participant weights post hoc, dose ranges were as follows: 0.012–0.015 mg/kg (1 mg cohort), 0.037–0.060 mg/kg (3.5 mg cohort), 0.059–0.093 mg/kg (5 mg cohort), 0.085–0.145 mg/kg (7.5 mg cohort), and 0.120–0.160 mg/kg (10 mg cohort). Participants received a single dose of MK-6194 or placebo via subcutaneous injection on day 1 and were followed for 29 days. PBMCs were collected for analysis on day –10, day 1 (predosing), day 8, day 15, and day 29. All participants provided written informed consent prior to study entry.

### MK-6194.

MK-6194 is an engineered protein consisting of modified recombinant human IL-2 (L118I, N88D, V69A, Q74P, C125S) fused to effector-function–ablated human IgG1 Fc, which augments molecule terminal elimination half-life. Modifications introduced into the IL-2 portion of MK-6194 increased the affinity for CD25, reduced the affinity for CD122, and enhanced manufacturability (15).

### In vitro stimulation of PBMCs.

Freshly isolated healthy human whole blood was collected in sodium heparin vacutainers. PBMCs were isolated using SepMate tubes (StemCell Technologies), washed with phosphate-buffered saline/2% fetal bovine serum (FBS), and dispensed into 96-well v-bottom plates at 200,000 cells per well in complete media (X-VIVO15 [Lonza] + 10% FBS [Omega]). MK-6194 or wild-type IL-2 (Cell Signaling Technologies) was added at final concentrations between 10 nM and 0.01 nM and incubated for 30 minutes at 37°C. Staining antibodies were added after fixation and permeabilization unless the epitope was fixation-sensitive. Thus, CD127, CD45RA, CD27, and CD16 were stained for 15 minutes before cells were fixed in CytoFix buffer (BD Biosciences), washed, permeabilized in Perm buffer III (BD Biosciences), washed, permeabilized with FOXP3 Perm buffer (BioLegend), washed, blocked in FOXP3 Perm buffer + 10% FBS, then immunolabeled with a cocktail of anti-CD25, anti-pSTAT5, anti-CD4, anti-CD56, anti-CD3, and anti-FOXP3. Antibody details are listed in Supplemental Table 2. Cells were labeled at room temperature in the dark for 30 minutes, washed twice in staining buffer, and resuspended in 500 μL staining buffer before flow cytometric analysis.

### In vivo assessment of Treg selectivity and expansion with humanized mice.

NSG mice (NOD.Cg-*Prkdc^scid^ Il2rg^tm1Wjl^*/SzJ; strain 005557, Jackson Laboratories) were engrafted with human CD34^+^ progenitor cells and rested for 9 days before being divided into 7 groups. One group was treated with a subcutaneous dose of sterile sucrose vehicle, and the other 6 groups were treated with a single subcutaneous dose of MK-6194. Groups were sacrificed on day 1, 3, 5, 6, 7, or 9 after dosing, and blood was collected into heparinized syringes. For dose response, blood was collected 7 days after treatment. Samples were immunolabeled with a cocktail of anti-CD25, anti-CD56, anti-CD127, anti-CD8, anti-CD45RA, anti-CD3, anti-CD16, anti-CD4, and anti-CD27 antibodies for 30 minutes at 37°C before fixing in 2 mL Fix Lyse (BD Biosciences). Antibody details are listed in Supplemental Table 3. Cells were washed and permeabilized with Perm III (BD Biosciences) on ice for 30 minutes, washed, permeabilized with FOXP3 Perm buffer (BioLegend) for 15 minutes in the dark at room temperature, washed, stained with an antibody cocktail for pSTAT5 and FOXP3 for 30 minutes, washed, and resuspended in staining buffer before flow cytometric analysis.

### In vivo assessment of Treg selectivity and expansion in cynomolgus monkeys.

Cynomolgus monkeys (*N* = 6) received a single subcutaneous injection of 0.5 mg/kg MK-6194. Whole blood was collected on day 7 after dosing and immunolabeled with either Panel 1 to measure conventional cell surface markers (CD3, CD159a, CD14, CD4, CD20, CD45, CD8) or Panel 2 to measure specific T cell populations (CD3, CD4, CD95, CD25, CD127, CD28). Antibody details are listed in Supplemental Table 4. For Panel 1, blood was incubated at room temperature, subjected to red blood cell lysis with ACK buffer, washed, resuspended in formaldehyde, and analyzed by flow cytometry. For Panel 2, blood was incubated at room temperature, subjected to red blood cell lysis with ACK buffer, washed, and then fixed and permeabilized using an eBioscience FOXP3/transcription factor staining buffer set. Cells were incubated with heat-inactivated serum, stained at room temperature for FOXP3 and Ki67, washed, resuspended, and analyzed by flow cytometry. Total T cells (CD3^+^), CD4^+^ T cells (CD3^+^CD4^+^), CD8^+^ T cells (CD3^+^CD8^+^), B cells (CD45^+^CD20^+^), and NK cells (CD45^+^CD159a^+^) were calculated from Panel 1. CD4^+^ T naive cells (CD3^+^CD4^+^CD28^+^ CD95^–^), CD4^+^ T central memory cells (CD3^+^CD4^+^CD28^+^CD95^+^), CD4^+^ T effector memory cells (CD3^+^CD4^+^CD28^–^CD95^+/–^), CD4^+^ Tconv cells (CD3^+^CD4^+^CD127^+^CD25^–^), and CD4^+^ Tregs (CD3^+^CD4^+^CD25^+^CD127^–^FOXP3^+^) were calculated from Panel 2. Absolute cell counts for each subset are reported according to a dual platform approach by multiplying the total lymphocyte concentration derived from an ADVIA 120 hematologic analyzer by the respective lymphocyte subset percentage derived from the flow cytometer.

### PBMC immunophenotyping by CyTOF.

Deep immunophenotyping was performed on previously frozen PBMCs from a subset of participants treated with MK-6194 or placebo. Details of the CyTOF panel are listed in Supplemental Table 2. In brief, PBMCs were thawed and barcoded with anti-CD45 antibodies, treated with the live/dead marker cisplatin, and labeled with antibodies against cell surface epitopes (Supplemental Table 5). Cells were then permeabilized with MaxPar Nuclear Antigen Staining buffer and Perm buffer, labeled with antibodies against intracellular antigens (Supplemental Table 5), washed, and fixed with 1.6% paraformaldehyde. Lastly, Cell-ID Intercalator-Ir was added. Samples were analyzed via CyTOF (Helios mass cytometer; Standard Bio); gating strategies for Tregs, Tconv, and NK cells are shown in Supplemental Figure 3. In addition to hierarchical gating, unbiased clustering with DISCOV-R was used to determine the effects of treatment on broad lymphocyte subsets (metaclusters) and Treg subsets (48). Phenotypic analysis of metaclusters is shown in Supplemental Figure 5, and phenotypic analysis of Treg subsets is shown in Supplemental Figure 8.

### Phosphoflow for pSTAT5.

To measure STAT5 activation, frozen PBMCs were thawed and cultured for 20 minutes at 37°C in complete RPMI-1640 media with or without 100 IU/mL recombinant human IL-2 (R&D Systems). Subsequently, cells were fixed and permeabilized with BD Phosflow Fix buffer I and BD Phosflow Perm buffer 3 (BD Biosciences). The following day, cells were stained with barcoded CD45 antibodies (Supplemental Table 6), which allowed stimulated and unstimulated PBMCs from each sample to be stained and analyzed in parallel. Samples were stained with the phosphoflow antibody panel, as shown in Supplemental Table 3, and acquired on an LSR Fortessa (BD Biosciences). Representative gating is shown in Supplemental Figure 7. Naive T cells were defined as CD45RA^+^CD27^+^ and memory T cells were defined as all non-naive cells.

### Assessing Treg selectivity of MK-6194 in PBMCs from people with autoimmune disease.

PBMCs from healthy individuals or people with T1D, SLE, or UC were thawed and rested at 37°C in complete RPMI-1640 media for 1 hour, followed by stimulation with either MK-6194 (90 nM) or recombinant human IL-2 (5 ng/mL) for 30 minutes. After stimulation, cells were collected, stained for viability, and fixed in 1× fix/perm buffer (Invitrogen) for 20 minutes. Cells were washed and fixed in 90% methanol for 30 minutes. Cells were subsequently washed and stained for all surface markers, FOXP3, and pSTAT5 in 1× permeabilization buffer (Invitrogen). Antibodies used are listed in Supplemental Table 7.

### Bulk and single-cell RNA-seq.

PBMCs were thawed and immediately immunolabeled and sorted by FACS for sequencing. Dead cells were excluded from all sorted cells by staining with Fixable Viability Dye eFluor 780 (eBioscience). For bulk RNA-seq, 250**–**500 CD4^+^CD127^lo^CD25^+^ Tregs were sorted directly into lysis buffer (representative sort gating in Supplemental Figure 9, antibody details in Supplemental Table 7), and cDNA was prepared using the SMART-Seq Ultra Low Input RNA Kit for Sequencing (Takara Bio). RNA-seq libraries were constructed using the NexteraXT DNA Library Preparation Kit (Illumina) with half the recommended volumes and reagents. Paired-end sequencing of pooled libraries was run on a NextSeq 2000 (Illumina) with 59-base reads and a target depth of 5 million reads per sample. After the run, base calling and demultiplexing were performed automatically on BaseSpace (Illumina) to generate FASTQ files. For scRNA-seq, 50,000–60,000 total CD122^+^ cells were sorted (representative sort gating in Supplemental Figure 6), and single-cell suspensions from each sample were loaded in a single channel of the 10× Chromium Controller (10× Genomics). Sequencing libraries were generated using the NextGEM Single Cell 5′ Kit v2. Sequencing of pooled libraries was carried out on a NextSeq 2000 sequencer (Illumina), with a target sequencing depth of 30,000 reads per cell. Cell Ranger (version 6.1.1; 10× Genomics) mkfastq was used to demultiplex and produce raw FASTQ files for downstream analyses.

### Analysis of bulk RNA-seq data.

FASTQ files were aligned to the human genome (GRCh38, Ensembl release 91) using STAR (v2.4.2a) (49). Gene counts were generated with HTSeq-count (v0.4.1) (50) in “intersection (nonempty)” mode, with a minimum alignment quality of 20 and all other parameters set to defaults. Quality metrics were compiled using PICARD (v1.134) (51), FASTQC (v0.11.3) (52), Samtools (v1.2) (53), and HTSeq-count (v0.4.1). Only samples with more than 1 million human-aligned reads and a median coefficient of variation coverage below 0.9 were retained. Genes were filtered to include those with a trimmed mean of M values (TMM) normalization count of at least 0.8 in at least 10% of samples and further filtered to retain only protein-coding genes. Normalized counts were transformed to log_2_(counts per million mapped reads), with observation-level weights applied using the voomWithQualityWeights function from the limma R package (v4.2.1) (54). The final data set consisted of 84 samples and 14,898 genes. DEGs were identified using the kimma R package (55) by linear mixed effects model for effect of treatment over time adjusting for median CV coverage: gene expression ~time*treatment + median CV coverage + (1|donor). Multiple testing correction was performed using the Benjamini-Hochberg procedure. Significant DEGs at an FDR of 0.2 or less for baseline to day 8 at 10 mg treatment were selected, and enrichment was run using GO biological processes gene sets using clusterProfiler (56). The significance of Treg and T effector cell signature changes over time was calculated using a linear mixed effects model, Expression ~time point + (1|donorId), using the lme4 (57) package in R. Treg gene sets were pulled from literature (19, 21) and enrichment was run using fgsea (58). For plotting purposes, ggplot2 (59) and ComplexHeatmap (60) packages were used in R (version 4.3.2).

### Analysis of scRNA-seq data.

All scRNA-seq analyses were performed in Monocle3 (61). Cells were filtered by percentage mitochondrial reads (<12.5%), as well as for outliers in sparsity (250) and excess (6000) of unique molecular identifier (UMI) counts. Gene expression values were log-transformed and the top 50 PCAs computed before reduction into the final UMAP. Leiden clustering at a resolution of 4 × 10^–5^ resulted in 10 distinct clusters. The top markers for each cluster were calculated by the top markers function; marker specificity and significance to cluster were established via a logistic regression of gene versus cluster assignment, and normalized marker gene expression was displayed on the UMAP. The proportion of each cluster in paired pre- and posttreatment samples were determined and significance calculated via the Wilcoxon test. Gene expression in each cluster of cells was then pseudobulked by donor and group condition, and DEGs between pre- and posttreatment samples were calculated in limma-voom (54).

### Transendocytosis assay.

Samples were thawed and CD4^+^ T cells were isolated by positive magnetic selection using human anti-CD4 MACS beads (Miltenyi Biotec) according to the manufacturer’s instructions. Isolated CD4^+^ T cells (5 × 10^4^) were added to 5 × 10^4^ NIH/3T3 cells (ATCC) that were stably transfected to express human CD80-mCherry and CD86-GFP fusion proteins in a 96-well flat-bottom plate in 200 mL of RPMI-1640 media containing 10% FBS, 1 mM sodium pyruvate, 10 mM HEPES, 2 mM L-glutamine, 50 U/mL penicillin, 50 mg/mL streptomycin, and 55 mM 2-mercaptoethanol. In some samples as indicated, 100 mg/mL of a blocking anti–CTLA-4 monoclonal antibody (clone BN13, BioXCell) was added. Cells were spun at 500*g* for 30 seconds to promote cell contact and cultured at 37°C for 6 hours. Bafilomycin A (Sigma-Aldrich) was added to a final concentration of 25 nM for the final 4 hours to prevent intracellular acidification and degradation of captured ligands. After incubation, the NIH/3T3 cells remained adherent, whereas CD4^+^ T cells were recovered from the cultures and stained for flow cytometric analysis of ligand capture by CD4^+^FOXP3^+^ Tregs and CD4^+^FOXP3^–^ Tconv cells (antibodies listed in Supplemental Table 7).

### Statistics.

Longitudinal Treg frequency data (Figure 2B) were analyzed using the mixed model for repeated measures (MMRM). MMRM included time points, dosing cohorts, and interaction of dosing cohorts by time points as fixed effects and baseline measurements as a covariate with random intercept. *P* values were then calculated to compare the differences of least square means between dosing cohorts versus placebo at each time point cross-sectionally. *P* values were adjusted for multiple comparison by FDR. R software version 4.3.2 and the nlme package were used to perform MMRM analyses. Other statistical analyses are described in figure legends and were conducted in GraphPad Prism, unless stated otherwise.

### Study approval.

All animal procedures were performed in accordance with the Animal Welfare Act Regulations. The human study was conducted according to principals of Good Clinical Practice and was approved by the site Institutional Review Board. All participants provided written informed consent prior to participation.

### Data availability.

Values for all data points in graphs are reported in the Supporting Data Values file. The data sharing policy, including restrictions, of Merck Sharp & Dohme LLC, a subsidiary of Merck & Co., Inc. is available at https://externaldatasharing-msd.com/ Data associated with phenotypic analyses of human Tregs from the phase Ia clinical trial will be made publicly available by the Immune Tolerance Network through their website, https://www.itntrialshare.org Bulk RNA-seq data of sorted Tregs and scRNA-seq data of sorted CD122^+^ cells from participants in the human single ascending dose clinical trial are available from the NCBI Gene Expression Omnibus under accession numbers GSE338670 (CD122^+^ cells) and GSE338655 (Tregs).

## Author contributions

The following authors contributed to study conceptualization: AK, SAL, SAS, BB, BL, NHS, DJC, ES, GAB, JFS, JLV, JV, KCB, KMH, KKT, KLO, LB, LAC, LK, NDK, RW, SA, and SS. The following authors contributed to study methodology: AK, SAL, SAS, BB, BL, NHS, DJC, ES, GAB, JFS, JLV, JV, KCB, KMH, KKT, KLO, LB, LAC, LK, NDK, RW, SA, and SS. The following authors contributed to study investigation: AK, SAL, SAS, BB, BL, NHS, DJC, ES, GAB, JFS, JLV, JV, KCB, KMH, KKT, KLO, LB, LAC, LK, NDK, RW, SA, and SS. The following authors contributed to data visualization: AK, SAL, SAS, BB, BL, NHS, DJC, ES, GAB, JFS, JLV, JV, KCB, KMH, KKT, KLO, LB, LAC, LK, MF, ML, and SS. The following authors contributed to funding acquisition: SAL, SAS, DJC, GAB, JFS, KC, LAC, NDK, RW, SA, and KMH. The following authors contributed to project administration: SAL, AS, DJC, ES, GAB, JLV, JV, KCB, KMH, KKT, KLO, LB, LAC, LK, NHS, NDK, and RW. The following authors contributed to project supervision: SAL, SAS, DJC, ES, GAB, JLV, JV, KCB, KMH, KKT, KLO, LB, LAC, LK, NHS, NDK, and RW. The following authors contributed to writing the original draft manuscript: AK, SAL, SAS, BB, BL, NHS, DJC, ES, GAB, JFS, JLV, JV, KCB, KMH, KKT, KLO, LB, LAC, LK, MF, ML, NDK, RW, SA, and SS. The following authors contributed to reviewing and editing the manuscript: AK, SAL, SAS, BB, BL, DJC, ES, GAB, JFS, JLV, JV, KCB, KMH, KKT, KLO, LB, LAC, LK, MF, ML, NHS, NDK, RW, SA, and SS.

## Conflict of interest

GAB, RW, KCB, and NDK are employees of Merck Sharp & Dohme LLC (MSD), a subsidiary of Merck & Co., Inc., and/or own stock in Merck & Co., Inc. SAS, JFS, and SA are former employees of MSD. BL, KKT, JLV, KLO, NHS, SA, and ES are previous employees of Pandion Therapeutics. There are granted patents owned by Pandion Therapeutics, Inc. (a wholly owned subsidiary of Merck & Co., Inc.) that claim MK-6194 and uses thereof. 20. The following US patents pertain to MK-6194: U.S. Patent No. 11,965,008; U.S. Patent No. 11,945,852; U.S. Patent No. 11,779,632; U.S. Patent No. 11,091,526; U.S. Patent No. 11,091,527; U.S. Patent No. 10,946,068. The following authors are listed as inventors: NHS, JLV, JV, ES, and KLO.

## Funding support

This work is, in part, the result of NIH funding and is subject to the NIH Public Access Policy. Through acceptance of this federal funding, the NIH has been given a right to make the work publicly available in PubMed Central.

- Merck Sharp & Dohme LLC, a subsidiary of Merck & Co., Inc.
- Work conducted by the Immune Tolerance Network was sponsored by the National Institute of Allergy and Infectious Diseases (NIAID), UM1AI109565 (recipient authors SAL, DJC, KMH, and LAC).
- Medical writing support was provided Helix, OPEN Health Communications, and funded by Merck Sharp & Dohme LLC, a subsidiary of Merck & Co., Inc., in accordance with Good Publication Practice guidelines (https://www.ismpp.org/gpp-2022).

## Supplementary Material

Supplemental data

Supporting data values

## Figures and Tables

**Figure 1:**
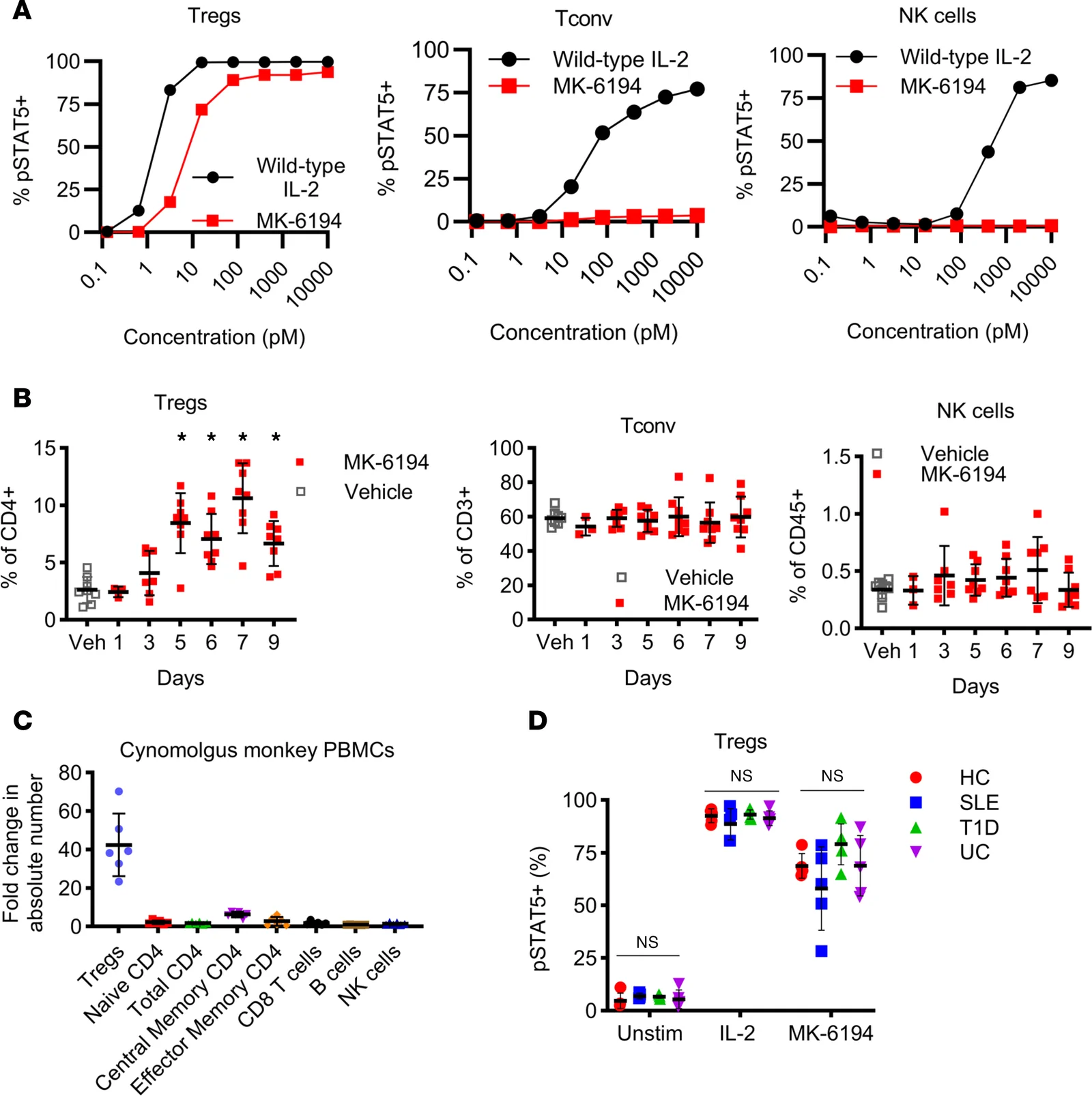
Selective and dose-dependent Treg expansion in vitro and in vivo following IL-2 mutein treatment. (**A**) Proportion of pSTAT5^+^ Tregs (CD3^+^CD4^+^FOXP3^+^CD25^+^CD127^–^), Tconv cells (CD3^+^CD4^+^FOXP3^–^), and NK cells (CD3^–^CD16^+^CD56^+^) following 30 minutes of stimulation with MK-6194 (red) or recombinant IL-2 (gray) (0.25 pM to 20 nM) using PBMCs isolated from healthy individuals. Data shown from 1 donor, representative of *n* = 5. (**B**) Frequency of Tregs (CD3^+^CD4^+^FOXP3^+^CD25^+^), Tconv (CD3^+^CD4^+^ FOXP3^–^), and NK cells (CD45^+^CD3^–^CD56^+^) from whole blood isolated on days 1, 3, 5, 6, 7, and 9 after treatment from NSG mice humanized with CD34^+^ hematopoietic stem cells. Mice were treated with 3 µg MK-6194 or vehicle (1-way ANOVA with Bonferroni’s multiple-comparison correction, *P < 0.05). Mean ± SD (*n* = 8). (**C**) Fold change in absolute number of PBMC subpopulations from cynomolgus monkeys 7 days after a single subcutaneous injection with 0.5 mg/kg MK-6194. Cell subsets were defined by flow cytometry as follows: Tregs (CD3^+^CD4^+^CD25^+^CD127^–^FOXP3^+^), naive CD4^+^ (CD3^+^CD4^+^CD28^+^CD95^–^), conventional CD4^+^ (CD3^+^CD4^+^CD127^+^CD25^–^), central memory (CD3^+^CD4^+^CD28^+^CD95^+^), effector memory (CD3^+^CD4^+^CD28^–^CD95^+/–^), CD8^+^ (CD3^+^CD8^+^), B cell (CD45^+^CD20^+^), and NK cell (CD45^+^CD159a^+^). Mean ± SD (*n* = 6). (**D**) Human PBMCs from healthy control donors (HC) or people with lupus (SLE), type 1 diabetes (T1D), or ulcerative colitis (UC) were stimulated in vitro with MK-6194 (90 nM, 7.6 ĩg/mL) or recombinant wild-type human IL-2 (5 ng/mL) for 30 minutes and pSTAT5 was assessed by flow cytometry. *N* = 5 per group. No significant differences between HC or autoimmune disease groups within a treatment condition by 2-way ANOVA. NS, not significant.

**Figure 2:**
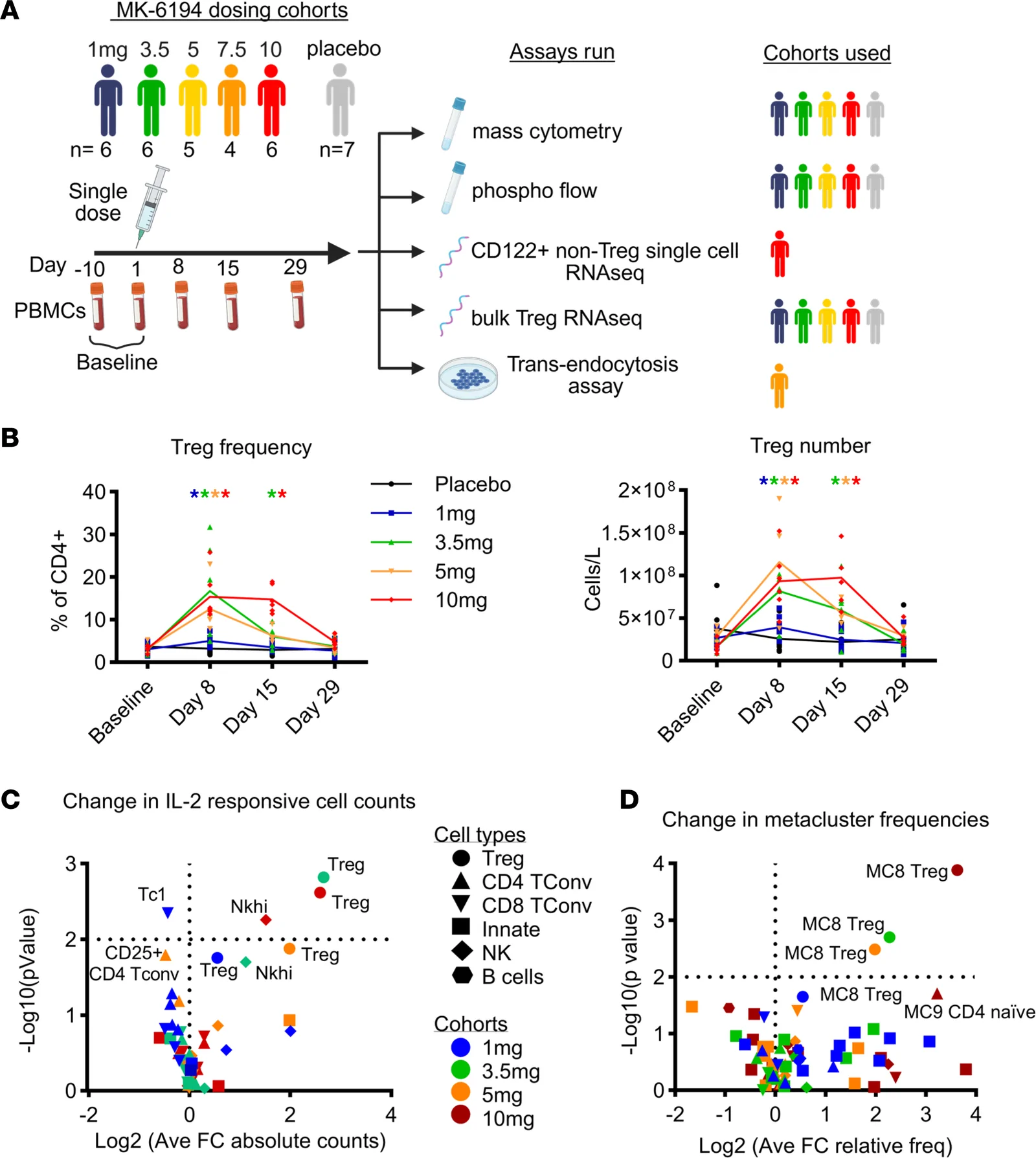
Selective and dose-dependent Treg expansion following IL-2 mutein treatment. (**A**) Study design of the human single ascending dose study, generated in BioRender. (**B**) Frequency among CD3^+^CD4^+^ T cells and absolute number of CD25^+^CD127^lo^FOXP3^+^ Tregs before and after treatment (gating in Supplemental Figure 3A). Absolute number based on lymphocyte complete blood counts obtained from clinical laboratory tests. *Significant after adjusting for multiple testing (*P* values in Supplemental Table 1), using mixed model for repeated measures (*n* as depicted in **A**); dose cohort annotated by asterisk color. (**C**) Log_2_-transformed average fold change in absolute cell number from baseline to day 8, using hypothesis-based gating of IL-2–responsive populations (gating in Supplemental Figure 3B). Paired 2-tailed *t* test, significance of 0.01 to account for multiple testing (*n* as in **A**). Participant-level data in Supplemental Figure 4. Cell type represented by shape; dose represented by color. “CD4 Tconv” group includes total CD4^+^ Tconv, CD25^+^CD4^+^ Tconv, Ki67^+^CD4^+^ Tconv, and Th1 subsets. “CD8 Tconv” group includes total CD8^+^ Tconv, CD25^+^CD8^+^ Tconv, Ki67^+^CD8^+^ Tconv, and Tc1 subsets. “Innate” group includes γδ T cells and innate lymphoid cell 2–like cells. “NK” group includes CD56^hi^ NK cells (NKhi) and CD56^lo^ NK (NKdim) cells. (**D**) Log_2_-transformed average fold change from baseline to day 8 in relative frequency of DISCOV-R automated CyTOF metaclusters. The 18 metaclusters were grouped into categories of Tregs, CD4^+^ Tconv, CD8^+^ Tconv, Innate, NK, and B cells as in **B**, represented by shape. Dose represented by color, as in **B**. Paired 2-tailed *t* test, significance of 0.01 to account for multiple testing (*n* as in **A**). Metacluster definitions and participant-level data shown in Supplemental Figure 5.

**Figure 3:**
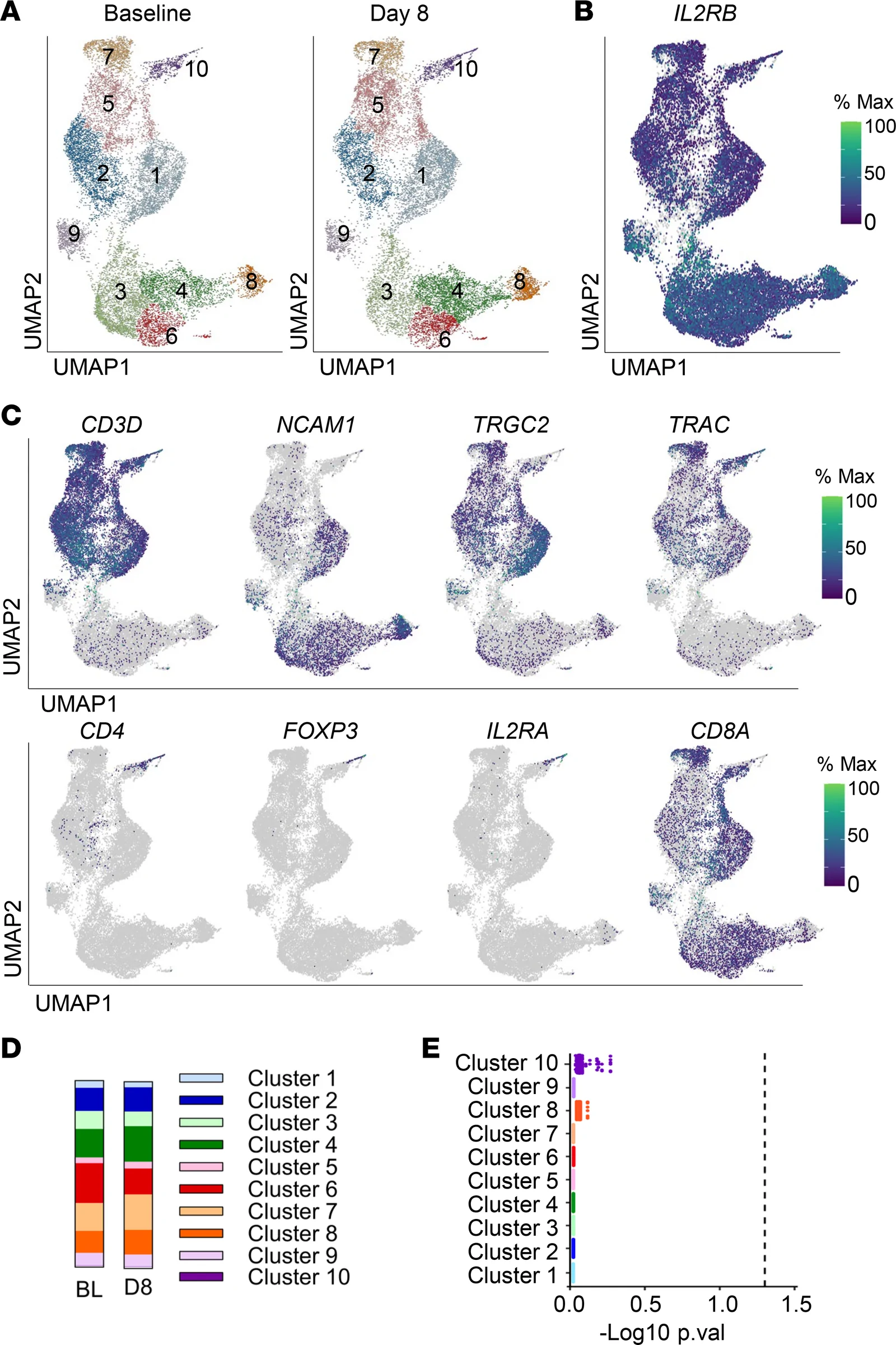
No changes in single-cell gene expression by CD122^+^ non-Tregs following IL-2 mutein treatment. (**A**) UMAP-based clustering of integrated scRNA-seq profiles of CD122^+^ cells sorted from either baseline or day 8 after MK-6194 treatment in the 10 mg dose cohort (*n* = 4). (**B**) Normalized expression of *IL2RB* (CD122) in each cell. (**C**) Normalized expression of the indicated lineage-specifying genes in each cell. (**D**) Comparison of cluster frequency between paired baseline (BL) and day 8 (D8) after MK-6194 treatment. No significant differences by the Wilcoxon test. (**E**) Differential gene expression from baseline versus day 8 after MK-6194 treatment in each pseudobulk cluster. Dashed horizontal line indicates threshold of *P* < 0.05, indicating no significant differences in gene expression.

**Figure 4:**
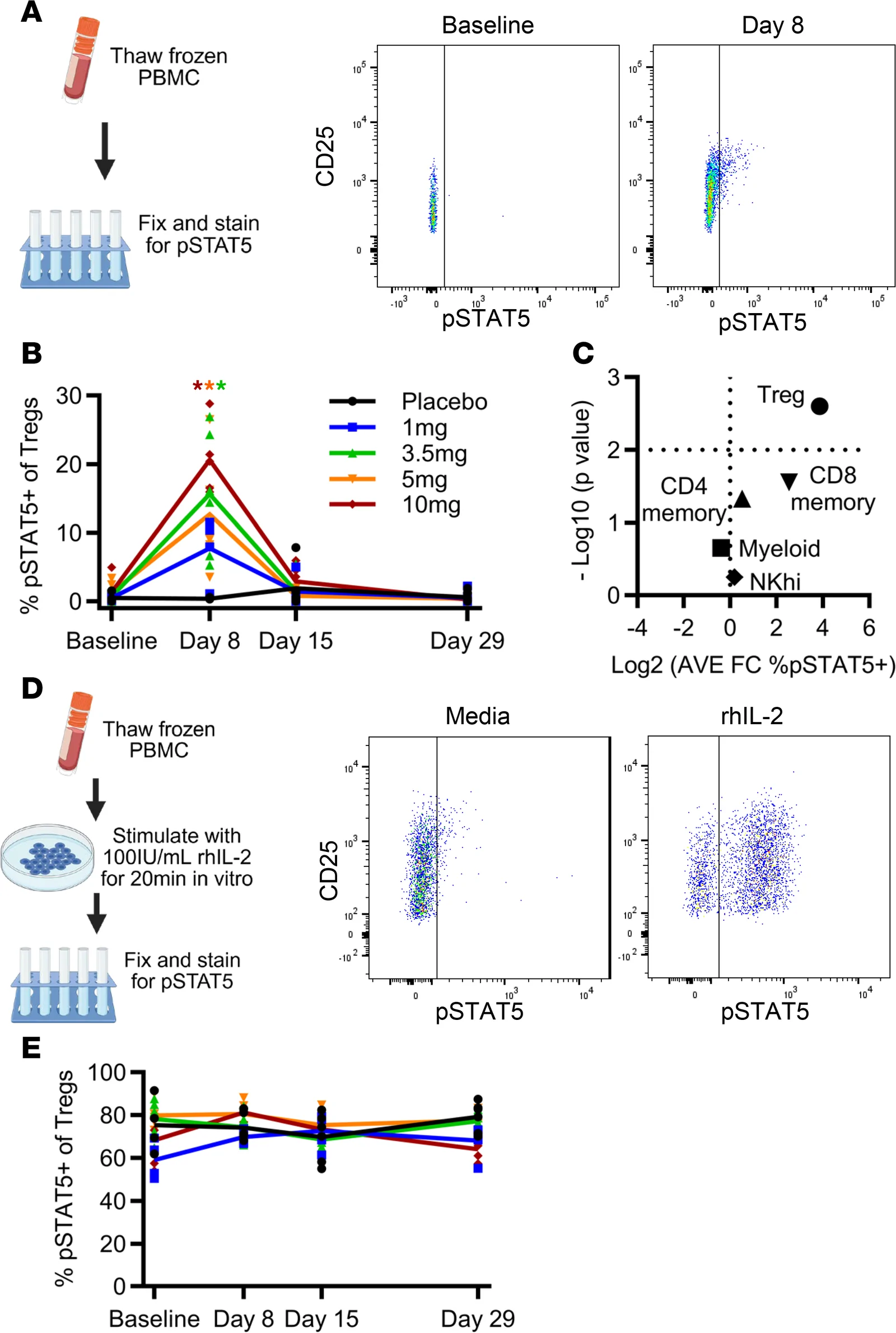
Prolonged, selective, dose-dependent pSTAT5 activation in Tregs following IL-2 mutein treatment in vivo. (**A**) Schematic and representative plots for ex vivo pSTAT5 staining, gated on CD4^+^CD25^+^CD127^–^ Tregs, for 1 participant at baseline versus day 8 after IL-2 mutein treatment (representative gating in Supplemental Figure 7A). Schematic generated in BioRender. (**B**) Frequency of pSTAT5^+^ cells among Tregs for all individuals, all time points (*n* as depicted in Figure 2A) when stained directly ex vivo without restimulation. Significance: *P < 0.05 versus placebo by mixed effects model with Dunnett’s correction for multiple comparisons; dose group reflected by color. (**C**) Log_2_-transformed average fold change in frequency of pSTAT5^+^ cells among various cell subsets from baseline to day 8 for 10 mg cohort (representative gating in Supplemental Figure 7A) when stained directly ex vivo without restimulation. Differences between baseline and day 8 determined using a Wilcoxon matched pairs signed rank test (*n* = 5). Cell types represented by shapes. (**D**) Schematic and representative plots for pSTAT5 staining of Tregs following in vitro restimulation with recombinant human IL-2 (rhIL-2) versus media alone for 1 sample. Schematic generated in BioRender. (**E**) Frequency of pSTAT5^+^ cells among Tregs for all cohorts, all doses following restimulation in vitro with rhIL-2; dose represented by color as in **A**. No significant differences by mixed effects model with Dunnett’s correction for multiple comparisons (*n* as depicted in Figure 2A).

**Figure 5:**
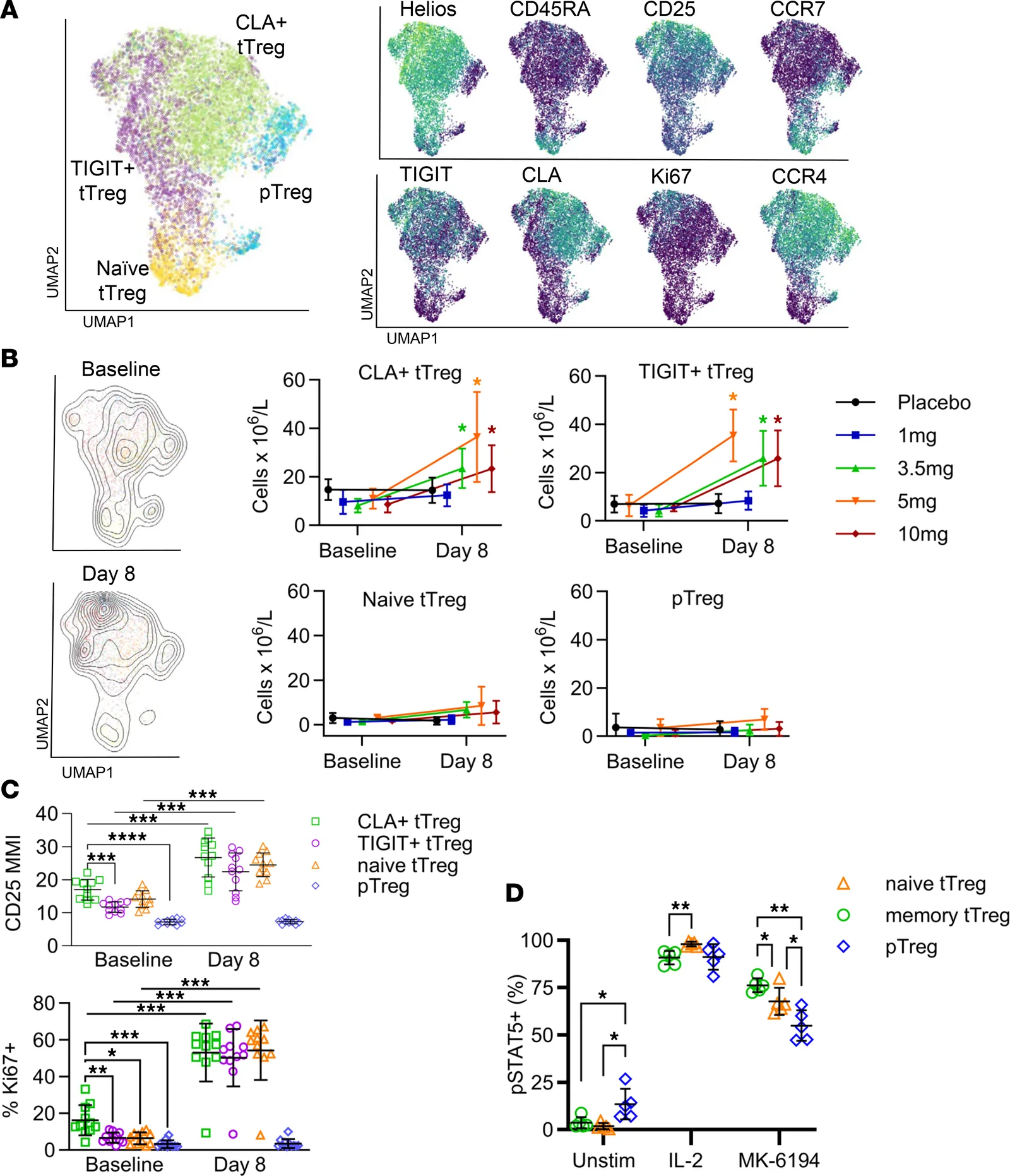
Preferential expansion of memory tTregs following IL-2 mutein treatment. (**A**) UMAP visualization of Treg (CD4^+^CD25^hi^FOXP3^+^) subclustering with heatmaps of selected subset-defining markers: green, high expression; blue, low expression. Includes integrated data from all cohorts at baseline and day 8. One cluster is defined as CD45RA^+^Helios^+^ naive tTregs (yellow), 2 clusters are defined as CD45RA^–^Helios^+^ memory tTregs (one with greater CLA expression, green; and the other with greater TIGIT expression, purple), and 1 cluster is defined as Helios^–^ pTregs (blue). (**B**) Comparisons of Treg cluster frequencies between baseline and day 8. Contour plots, average ± SD of absolute counts for all cohorts across all individuals. CLA^+^ tTregs (time, *P* < 0.0001; dose, *P* = 0.0394) and TIGIT^+^ tTregs (time, *P* < 0.0001; dose, *P* = 0.0001) were significantly different by 1-way ANOVA. Mean ± SD of absolute cell counts for each Treg subset at baseline versus day 8. Dose indicated by color, Wilcoxon’s rank paired test. *P < 0.05. *N* as depicted in Figure 2A. (**C**) Comparison of CD25 mean metal intensity (MMI) and frequency of Ki67^+^ cells for all 3 tTreg clusters and 1 pTreg cluster. Data from the 3.5 mg and 10 mg cohorts combined. Repeated measures 1-way ANOVA with Bonferoni’s correction for multiple comparisons. **P < 0.01; ***P < 0.001; ****P < 0.0001. NS, no significant differences between any 2 groups (*n* = 11–12). (**D**) Healthy control human PBMCs were stimulated in vitro with MK-6194 (90 nM, 7.6 μg/mL) or recombinant wild-type human IL-2 (5 ng/mL) for 30 minutes, followed by flow cytometric analysis for induction of pSTAT5. Naive tTreg = CD45RA^+^Helios^+^, memory tTreg = CD45RA^–^Helios^+^, pTreg = CD45RA^–^Helios^–^ (*n* = 5). *P < 0.05; **P < 0.01 by 2-way ANOVA with Tukey’s multiple-comparison test.

**Figure 6:**
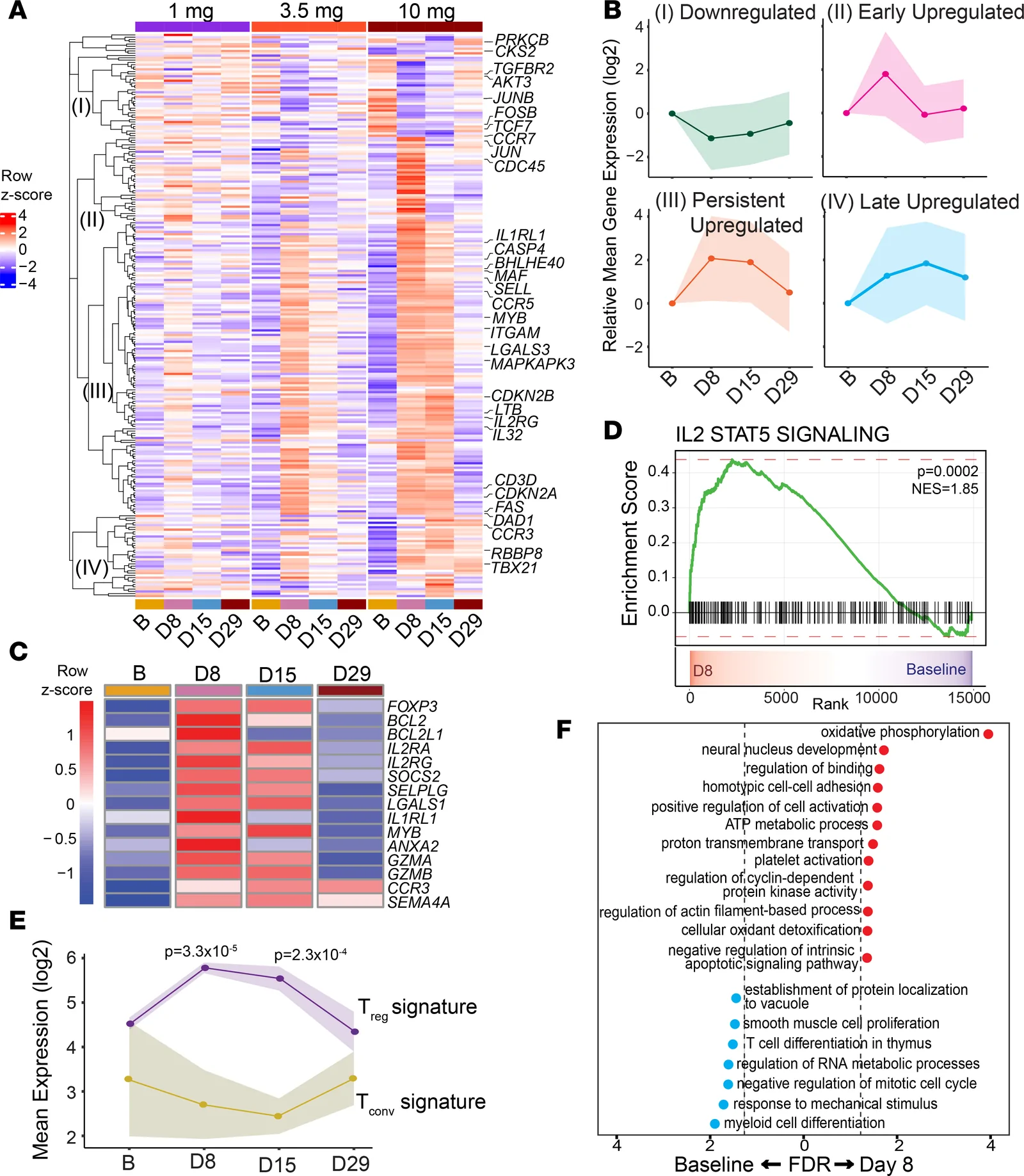
Transient activation-associated transcriptional changes in circulating Tregs following IL-2 mutein treatment. (**A**) Heatmap showing *z*-scored average expression of all DEGs in sorted Tregs (CD4^+^CD25^+^CD127^lo^) following MK-6194 treatment shown across all time points in the following dosing cohorts: 1 mg (*n* = 5), 3.5 mg (*n* = 6), and 10 mg (*n* = 4). Dendrogram shows 4 distinct patterns of gene expression change following treatment. B, baseline. (**B**) Graphs show log_2_-normalized mean gene expression of all genes in each gene expression category as indicated in **A** in the 10 mg dose cohort. Shaded region indicates 95% confidence interval. (**C**) Heatmap showing *z*-scored average expression of select STAT5-regulated genes at each time point in the 10 mg dose cohort. (**D**) Gene set enrichment analysis showing significant enrichment of genes in the Hallmark IL2 STAT5 signaling gene set in Tregs 8 days after MK-6194 treatment in the 10 mg dose cohort compared with baseline samples. (**E**) Graph showing log_2_ mean expression of Treg and Tconv signature genes in sorted Tregs at each time point in the 10 mg dose cohort. Significance was calculated using a linear mixed effect model. (**F**) Gene Ontology term enrichment analysis of DEGs between baseline and day 8 posttreatment samples from the 10 mg dose cohort. Dashed lines indicate FDR significance threshold.

**Figure 7:**
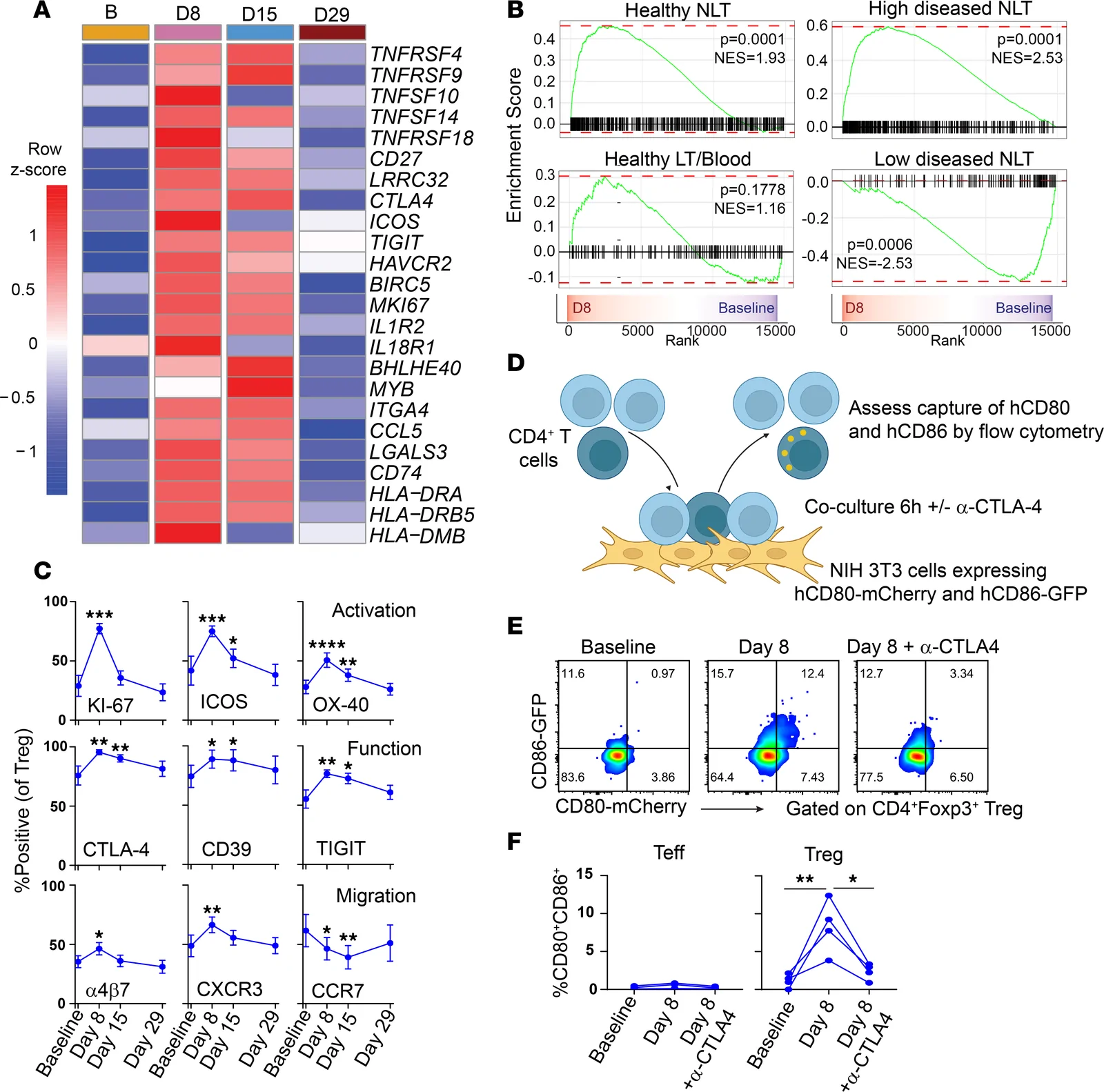
Increased expression of markers of Treg activation, function, and tissue-homing potential following IL-2 mutein treatment. (**A**) Heatmap showing *z*-scored average expression of select Treg activation genes at each time point in the 10 mg dose cohort (*n* = 4) by bulk RNA-seq of sorted CD4^+^CD25^+^CD127^lo^ Tregs. (**B**) Gene set enrichment analysis showing enrichment of public consensus Treg gene sets in Tregs from baseline versus day 8 (D8) following 10 mg MK-6194 treatment. The public consensus Treg gene sets “healthy non-lymphoid tissue (NLT)” and “high in diseased NLT” are enriched on day 8 following MK-6194 treatment, and the gene set “low in diseased NLT” is enriched at baseline. No significant enrichment of the “Healthy LT/Blood” gene set at either time point. (**C**) Percentage of Tregs (CD4^+^CD25^+^CD127^lo^FOXP3^+^) expressing the indicated markers at each time point in the 10 mg dose cohort as measured by CyTOF (mean ± SD, *n* = 6). Significant changes relative to baseline frequencies were calculated by 2-way ANOVA with Dunnett’s correction for multiple comparisons. *P < 0.05, **P < 0.01, ***P < 0.001, ****P < 0.0001. (**D**) Schematic of CTLA-4 transendocytosis assay. (**E**) Representative flow cytometric analysis of CD80-mCherry and CD86-GFP capture by Tregs (CD4^+^FOXP3^+^) from baseline and day 8 after 7.5 mg MK-6194 treatment as indicated (*n* = 4). Addition of blocking anti–CTLA-4 antibody demonstrates specificity of capture. (**F**) Graphs showing percentage of CD80^–^mCherry^+^CD86^–^GFP^+^ Tconv cells and Tregs in paired baseline and day 8 after 7.5 mg MK-6194 treatment samples (*n* = 4). Significance calculated by repeated-measures 1-way ANOVA with Holm-Šídák multiple-comparison test. **P < 0.01; *P < 0.05.

## Version 1 09/17/2026

In-Press Preview

## Version 2 10/22/2026

Electronic publication
