# Supplementary material for: IL-2 mutein selectively expands thymic memory Tregs with increased transendocytosis in healthy participants
Source: JCI Insight. 2026 Sep 17;11(20):e193744. doi: 10.1172/jci.insight.193744 (PMC13726557; doi:10.1172/jci.insight.193744)
Supplement: Supplementary file 1 [file jciinsight-11-193744-s038.pdf]

Supplementary Materials

IL-2 mutein selectively expands thymic memory Tregs with increased suppressive function in healthy participants

Laura A. Cooney, Mitch Fahning, Liliane Khoryati, Anna Kus, Sheila Scheiding, Lori Blanchfield, Matt Lawrence, Basilin Benson, Kristina M. Harris, Gretchen A. Baltus, Shiuli Agarwal, Richard Wnek, Johannes F. Scheid, Kiki Cunningham-Bussel, Nancy D. Kim, Aubrey Stoch, Jyothsna Visweswaraiah, Nathan Higginson-Scott, Katalin Kis-Toth, Joanne L. Viney, Kevin L. Otipoby, Erik Sampson, Bridget Larkin, Daniel J. Campbell, S. Alice Long

SUPPLEMENTARY FIGURES

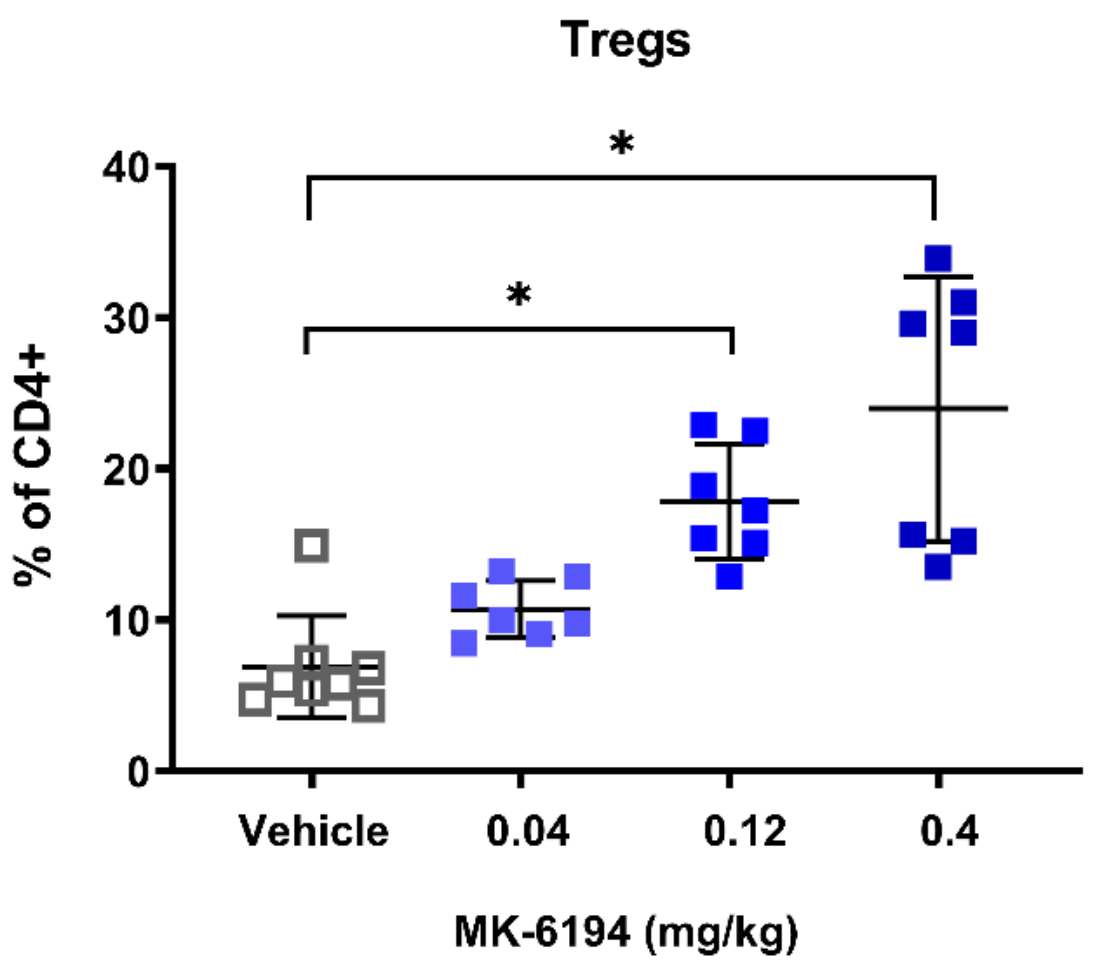

Figure S1. Dose response of MK-6194 in humanized mice. Humanized mice received a single injection of MK-6194 at 0.04, 0.12, or 0.4 mg/kg or vehicle. After 7 days, blood was collected and Treg frequency was assessed by flow cytometry.

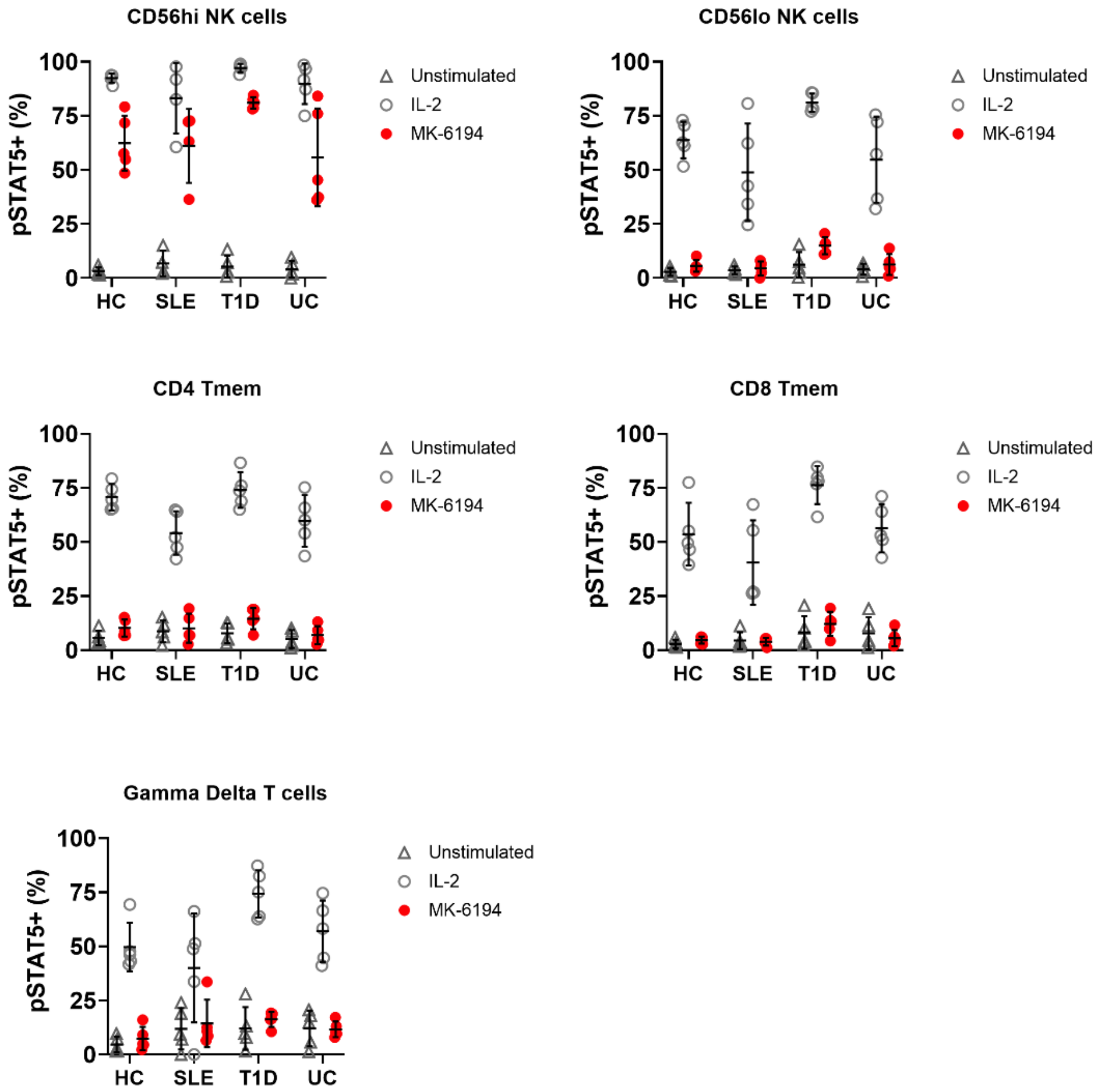

Figure S2. MK-6194 demonstrates similar selectivity during in vitro stimulation of human PBMCs from healthy controls versus patients with autoimmune disease.

PBMCs from healthy controls (HC) or patients with lupus (SLE), type 1 diabetes (T1D), or ulcerative colitis (UC) were stimulated in vitro with MK-6194 (7.6ug/mL) or recombinant wildtype human IL-2 (5ng/mL) for 30 minutes and pSTAT5 was assessed by flow cytometry. N = 5 per group.

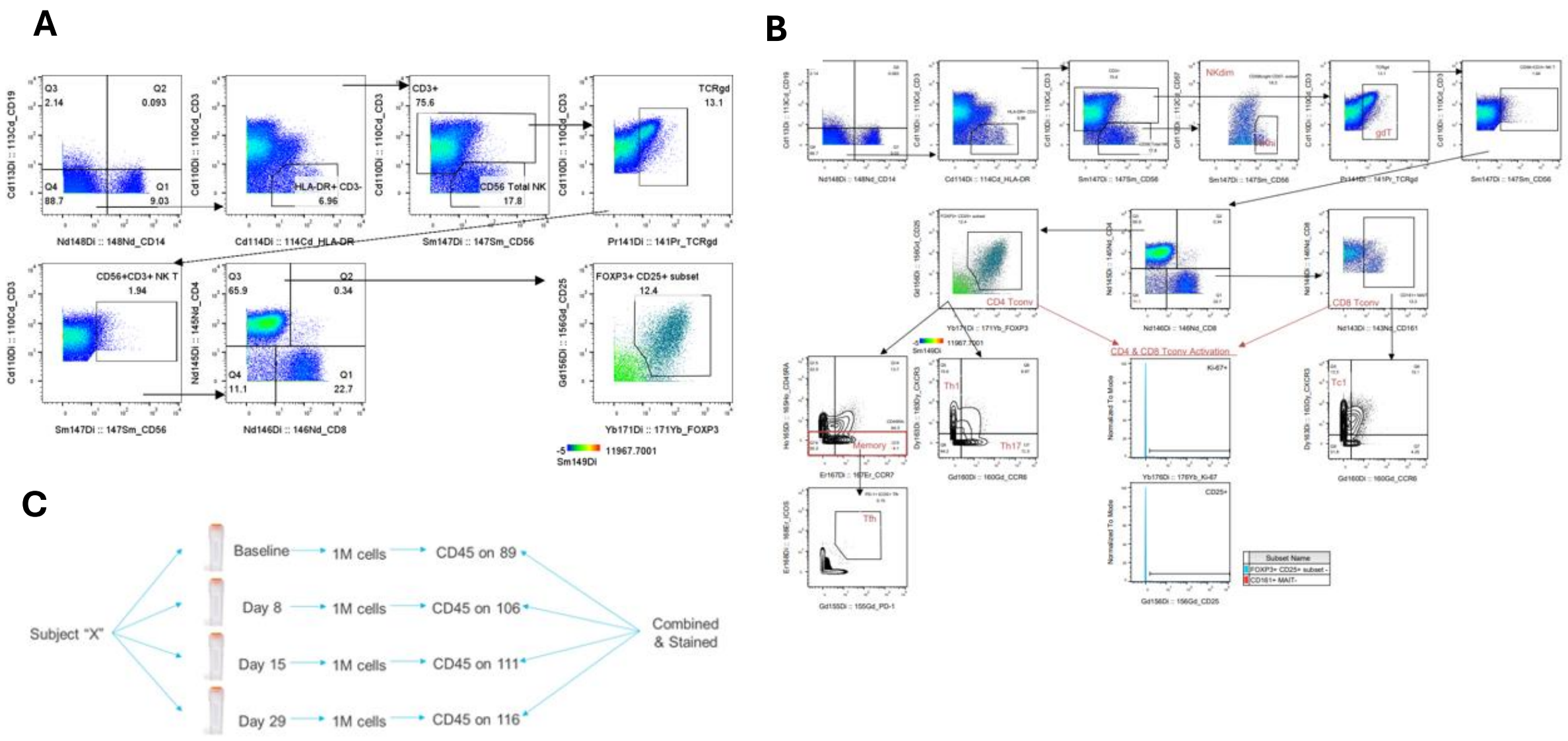

Fig. S3. Representative subset gating by cytometry by time-of-flight. (**A**) Representative Treg gating. (**B**) Representative gating of other potentially IL-2–responsive cell subsets, including NK^{hi}, NK^{dim}, $CD4^{+}$, and $CD8^{+}$ Tconv, Th1, and Tc1. (**C**) Diagram of CD45 barcoding scheme to allow for simultaneous staining and acquisition of all samples from a given participant.

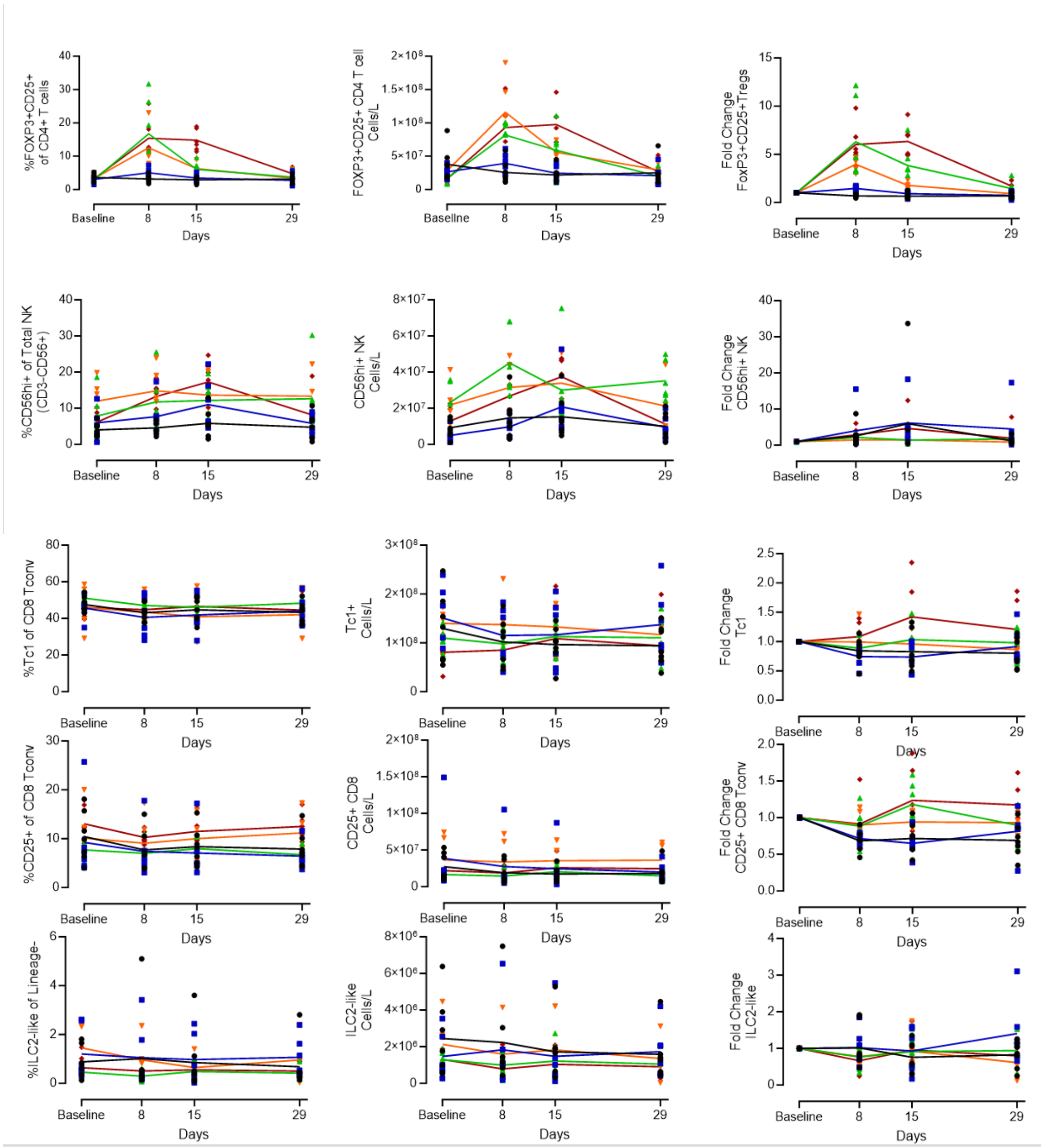

Fig. S4. Relative frequency, absolute frequency, and fold change in absolute frequency of predicted IL-2–responsive cell subsets, including Treg, CD56^{hi} NK cells, Tc1 subset of CD8^{+} T cells, CD25^{+} subset of CD8^{+} T cells, and innate lymphoid cell 2 (ILC2)-like cells from cytometry by time-of-flight analyses of all participants and all time points. Dose represented by color, as in other figures. Black = placebo, blue = 1mg, green = 3.5mg, orange = 5mg, red = 10mg. Note that this data is also shown in Figure 2B and summarized in Figure 2C (Day 8 fold change in absolute counts).

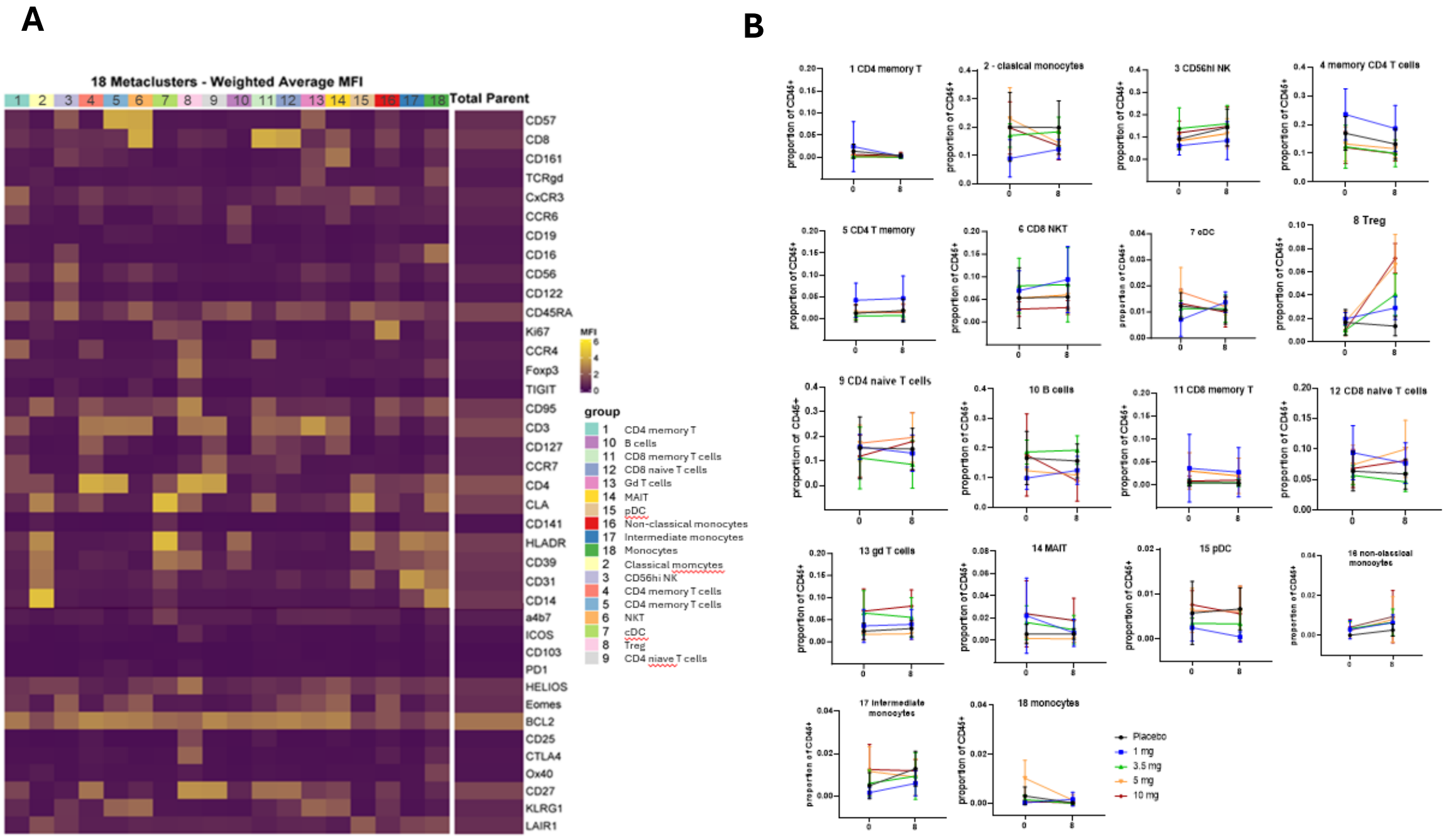

Fig. S5. DISCOV-R metaclusters. (**A**) Weighted-average expression of all cytometry by time-of-flight markers across all 18 metaclusters, expressed as mean staining intensity (MFI). (**B**) Relative frequency of all 18 metaclusters as a percentage of total CD45^{+} cells at baseline and Day 8 across all dosing cohorts. Note that this data is also summarized in Figure 2D as fold change.

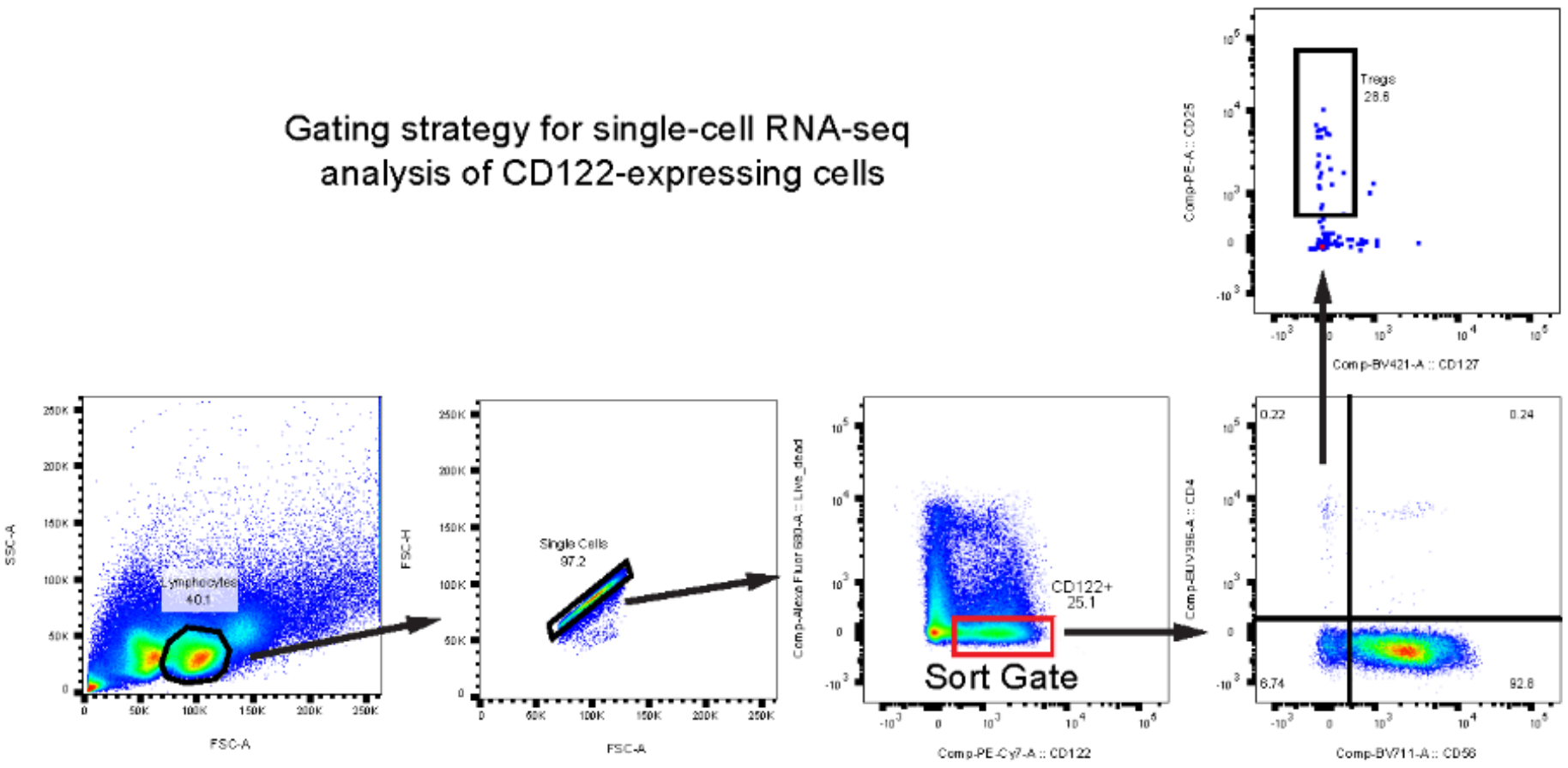

Fig. S6. Gating strategy for sorting CD122^{+} cells for single-cell RNA sequencing. Additional flow analysis of CD122^{+} cells demonstrates that most cells are CD56^{+}, with very few CD4^{+} Tconv or CD4^{+}CD25^{+}CD127^{lo} Tregs.

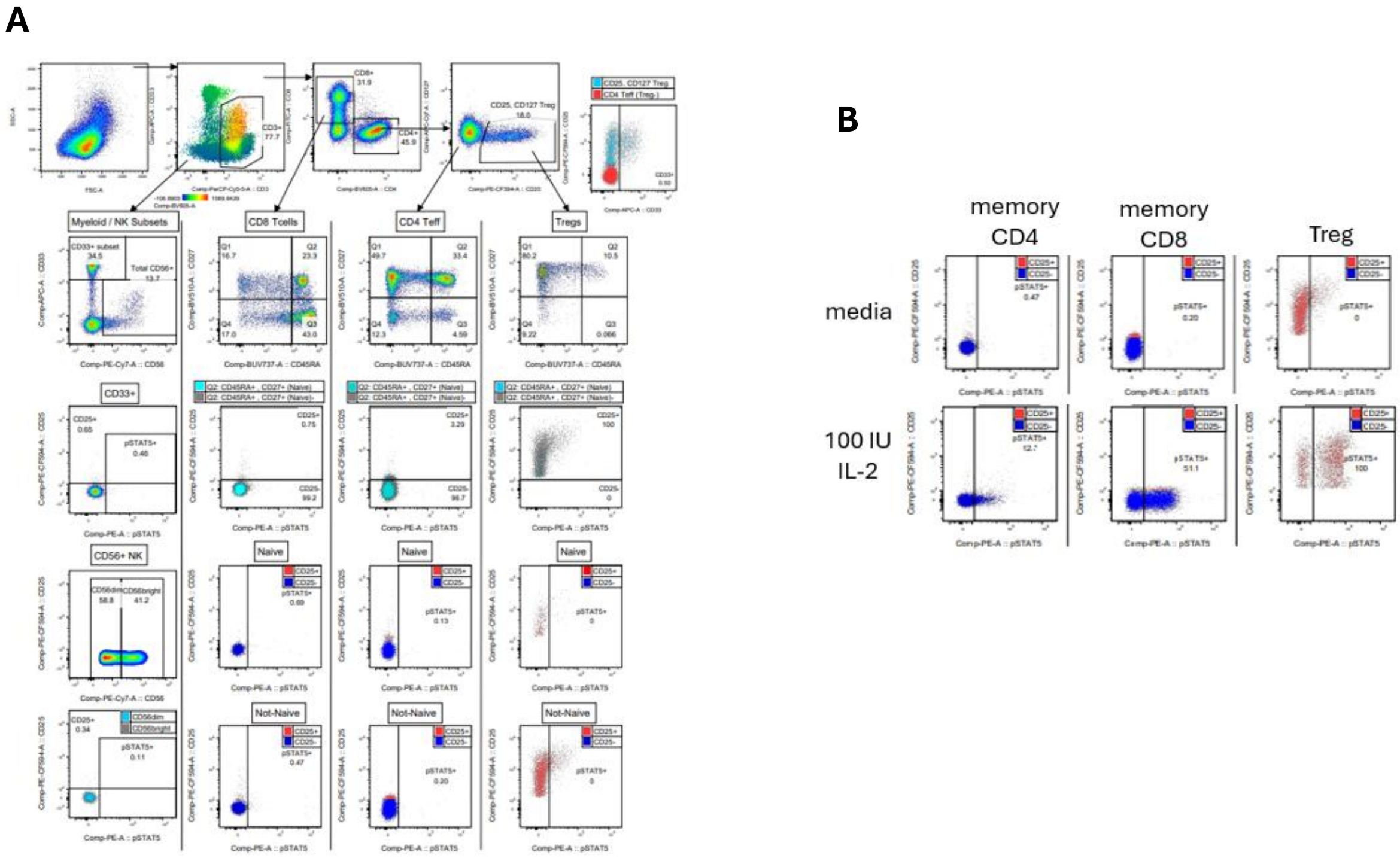

Fig. S7. Representative gating and staining for phosphoflow experiments. (A) Representative gating for Tregs and other potentially IL-2–responsive cell subsets, such as NK, $CD4^+$ Tconv, and $CD8^+$ T cells. **(B)** Representative pSTAT5 staining in Tregs with and without in vitro stimulation with recombinant human IL-2.

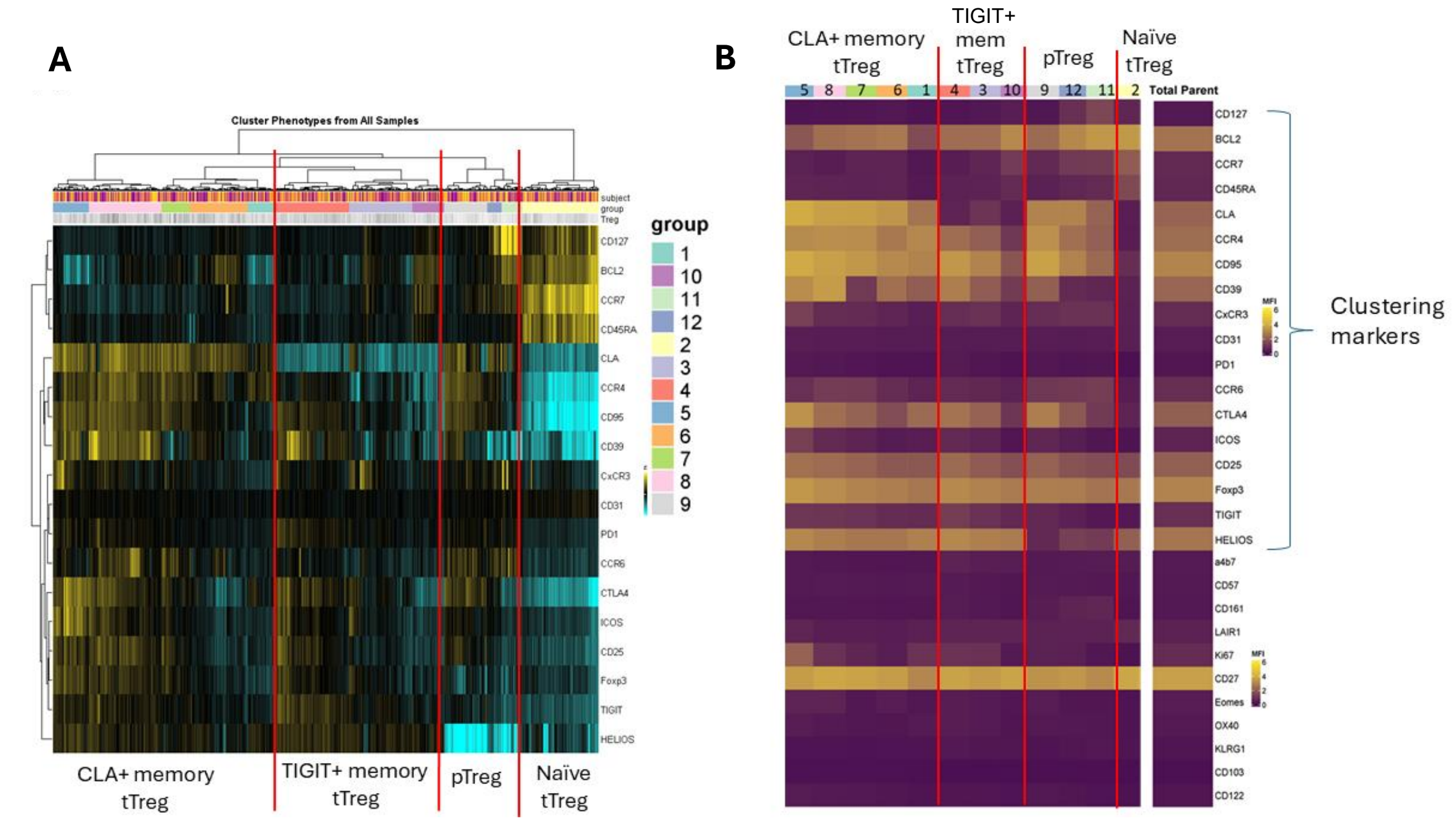

Fig. S8. Subclustering of Tregs based on cytometry by time-of-flight (CyTOF). (**A**) *Z*-scored expression of each of the 18 markers used for Treg subclustering in each sample, with dendrogram demonstrating relationship between clusters. Original 12 clusters were grouped into four subsets: CLA^{+} memory tTreg, $TIGIT^{+}$ memory tTreg, naïve tTreg, and pTreg. (**B**) Weighted-average staining intensity of 18 markers used for Treg subclustering, in addition to other markers of interest, across the 12 original clusters.

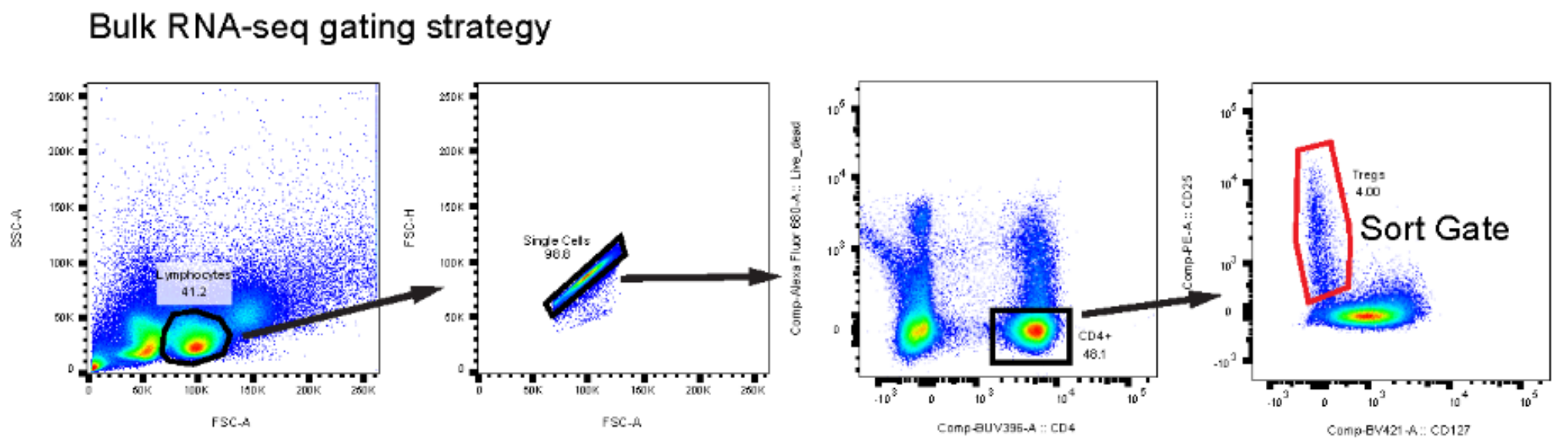

1

Fig. S9. Representative Treg sorting strategy for bulk RNA sequencing. Tregs were sorted as $CD4^{+}CD127^{lo}CD25^{+}$.

SUPPLEMENTARY TABLES

Table S1. P-values for Fig. 2B.

Dose	Time point	Treg % of CD4	Treg absolute #
1 mg vs. placebo	Day 8	0.018*	0.203
	Day 15	1	1
	Day 29	1	1
3.5 mg vs. placebo	Day 8	4.27E-09*	3.64E-06*
	Day 15	2.12E-04*	1.27E-04*
	Day 29	0.928	1
5 mg vs. placebo	Day 8	2.26E-07*	7.33E-06*
	Day 15	0.002*	0.022*
	Day 29	1	1
10 mg vs. placebo	Day 8	5.02E-09*	1.20E-06*
	Day 15	2.08E-09*	2.61E-07*
	Day 29	0.111	0.274

Mixed model for repeated measures; P-value adjusted for multiple comparisons using false discovery rate, as described in Statistical methods section. $^*P < 0.05$.

Table S2. Antibodies used for human PBMC stimulation in vitro (Fig. 1A)

Target	Format	Clone	Vendor	Catalog #
CD127	PE-Cy7	A019D5	BioLegend	351320
CD45RA	BV510	HI100	BioLegend	304142
CD27	BV711	L128	BD Biosciences	563167
CD16	BV650	3G8	BioLegend	302042
**CD25**	PE	M-A251	BD Biosciences	555432
**CD3**	BV421	UCHT1	BioLegend	300434
**FoxP3**	Alexa Fluor 647	259D/C7	BD Biosciences	560045
**pSTAT5**	Alexa Fluor 488	C71E5	Cell Signaling Technologies	3939
**CD4**	PerCP-Cy5.5	RPA-T4	BioLegend	300530
**CD56**	BV785	5.1H11	BioLegend	362550

Those in bold were stained post fix/perm.

Table S3. Antibodies used for analysis of humanized mice (Fig. 1B)

Target	Format	Clone	Vendor	Catalog #
CD25	PE	M-A251	BD Biosciences	555432
CD56	PerCP-Cy5.5	5.1H11	BioLegend	362506
CD127	PE-Cy7	A019D5	BioLegend	351320
CD8	APC-Cy7	RPA-T8	BioLegend	301016
CD45RA	BV510	HI100	BioLegend	304142
CD3	BV421	UCHT1	BioLegend	300434
CD16	BV650	3G8	BioLegend	302042
CD4	BV711	OKT4	BioLegend	317440
CD27	BV786	L128	BD Biosciences	563327
**pSTAT5**	Alexa Fluor 488	C71E5	Cell Signaling Technology	3939
**FoxP3**	Alexa Fluor 647	259D/C7	BD Biosciences	560045

Those in bold were stained post fix/perm.

Table S4. Antibodies used for cynomolgus monkey experiments (Fig. 1C)

Target	Format	Clone	Vendor
CD3	Alexa Fluor 488	SP34-2	BD Biosciences
CD159a	PE	Z199	Miltenyi Biotec
CD14	PerCP-Cy5.5	M5E2	BD Biosciences
CD4	PE-Cy7	L200	BD Biosciences
CD20	V450	L27	BD Biosciences
CD45	V500	D058-1283	BD Biosciences
CD8	APC-H7	SK1	BD Biosciences
CD3	PerCP-Cy5.5	SP34-2	BD Biosciences
CD4	APC-H7	L200	BD Biosciences
CD95	BV510	DX2	BD Biosciences
CD25	PE	M-A251	BD Biosciences
CD127	APC	MB15-18C9	Miltenyi Biotec
CD28	PE-Cy7	CD28.2	BD Biosciences
**FoxP3**	V450	259D/C7	BD Biosciences
**Ki67**	Alexa Fluor 488	B56	BD Biosciences

Those in bold were stained post fix/perm.

Table S5. Antibodies used for human PBMC CyTOF (Figs. 2 & 5)

Label	Target	Clone	Vendor
89Y	Barcode: Pan CD45	HI30	Standard BioTools
106Cd	Barcode: Pan CD45	HI30	Standard BioTools
110Cd	CD3	UCHT1	Conjugated by BRI
111Cd	Barcode: Pan CD45	HI30	Standard BioTools
112Cd	CD57	HCD57	Conjugated by BRI
113Cd	CD19	HIB19	Conjugated by BRI
114Cd	HLADR	L243	Conjugated by BRI
116Cd	Barcode: Pan CD45	HI30	Standard BioTools
141Pr	TCRgd	B1	Conjugated by BRI
142Nd	Ox40	Act35	Standard BioTools
143Nd	CD161	4B4-1	Conjugated by BRI
144Nd	CD31	WM59	Standard BioTools
145Nd	CD4	RPA-T4	Standard BioTools
146Nd	CD8	RPA-T8	Standard BioTools
147Sm	CD56	NCAM16.2	Conjugated by BRI
148Nd	CD14	RM052	Standard BioTools
149Sm	CD127	A019D5	Standard BioTools
150Nd	LAIR1	DX26	Conjugated by BRI
151Eu	**HELIOS**	22F6	Conjugated by BRI
152Sm	**CTLA4**	BNI3	Conjugated by BRI
153Eu	CCR4	L291H4	Standard BioTools
154Sm	TIGIT	MBSA	Standard BioTools
155Gd	PD1	EH12.2H7	Standard BioTools
156Gd	CD25	M-A251	Conjugated by BRI
158Gd	CD27	L-128	Standard BioTools
159Tb	**BCL2**	bcl-2/100	Conjugated by BRI
160Gd	CCR6	G034E3	Conjugated by BRI
161Dy	CD39	A1	Conjugated by BRI
162Dy	**Eomes**	WD1928	Conjugated by BRI
163Dy	CxCR3	G025H7	Standard BioTools
164Dy	CD95	DX2	Standard BioTools
165Ho	CD45RA	HI100	Conjugated by BRI
166Er	CD141	M80	Standard BioTools
167Er	CCR7	G043H7	Standard BioTools
168Er	ICOS	C398.4A	Standard BioTools
169Tm	KLRG1	REA	Conjugated by BRI
170Er	CD122	Tu27	Standard BioTools
171Yb	**Foxp3**	259D/C7	Conjugated by BRI

173Yb	CD103	Ber-ACT8	Conjugated by BRI
174Yb	α4β7	AD5-17F6	Conjugated by BRI
175Lu	CLA	HECA-452	Conjugated by BRI
176Yb	**Ki67**	Ki-67	Conjugated by BRI
209Bi	CD16	3G8	Standard BioTools

Those in bold are intracellular antigens.

BRI, Benaroya Research Institute

Table S6. Antibodies used for phosphoflow of clinical trial samples (Fig. 4)

Target	Format	Clone	Vendor
Pan CD45 (Barcode)	BUV395	HI30	BD Biosciences
Pan CD45 (Barcode)	BV785	HI30	Biolegend
Pan CD45 (Barcode)	BV421	HI30	Biolegend
**pSTAT5** (pY694)	PE	A17061B.Rec	Biolegend
CD127	APCef780 (H7)	RDR5	eBioscience
CD8	FITC	RPA-T8	BioLegend
CD4	BV605	RPA-T4	BD Biosciences
CD27	BV510	L128	BD Biosciences
CD45RA	BUV737	Hl100	BD Biosciences
CD25	PE-CF594	M-A251	BD Biosciences
CD3	PerCP-Cy5.5	UCHT1	BioLegend
CD33	APC	P67.6	BioLegend
CD56	PE-CY7	5.1H11	BioLegend

Those in bold are intracellular antigens.

Table S7. Antibodies used for cell sorting and transendocytosis assay

Target	Format	Clone	Vendor	Catalog #
CD4	BUV395	RPA-T4	BD Biosciences	564724
CD127	BV421	A019D5	Biolegend	351310
CD56	BV711	HCD56	Biolegend	318336
CD25	PE	M-A251	Biolegend	356134
CD122	PE-Cy7	TU27	Biolegend	339014
CD25	BV421	BC96	Biolegend	302629
CD4	BV510	OKT4	Biolegend	317444
**Foxp3**	PE	206D	Biolegend	320108
Fixable Viability Dye	efluor780		ThermoFisher	65-0865-18

Those in bold are intracellular antigens.
